# Mechanisms of noncovalent β subunit regulation of Na_V_ channel gating

**DOI:** 10.1085/jgp.201711802

**Published:** 2017-08-07

**Authors:** Wandi Zhu, Taylor L. Voelker, Zoltan Varga, Angela R. Schubert, Jeanne M. Nerbonne, Jonathan R. Silva

**Affiliations:** 1 Department of Biomedical Engineering, Washington University in St. Louis, St. Louis, MO; 2 Department of Developmental Biology, Washington University in St. Louis, St. Louis, MO; 3 Department of Internal Medicine, Washington University in St. Louis, St. Louis, MO; 4 MTA-DE-NAP B Ion Channel Structure-Function Research Group, RCMM, University of Debrecen, Debrecen, Hungary

## Abstract

Voltage-gated Na^+^ (Na_V_) channels comprise a macromolecular complex whose components tailor channel function. Key components are the non-covalently bound β1 and β3 subunits that regulate channel gating, expression, and pharmacology. Here, we probe the molecular basis of this regulation by applying voltage clamp fluorometry to measure how the β subunits affect the conformational dynamics of the cardiac Na_V_ channel (Na_V_1.5) voltage-sensing domains (VSDs). The pore-forming Na_V_1.5 α subunit contains four domains (DI–DIV), each with a VSD. Our results show that β1 regulates Na_V_1.5 by modulating the DIV-VSD, whereas β3 alters channel kinetics mainly through DIII-VSD interaction. Introduction of a quenching tryptophan into the extracellular region of the β3 transmembrane segment inverted the DIII-VSD fluorescence. Additionally, a fluorophore tethered to β3 at the same position produced voltage-dependent fluorescence dynamics strongly resembling those of the DIII-VSD. Together, these results provide compelling evidence that β3 binds proximally to the DIII-VSD. Molecular-level differences in β1 and β3 interaction with the α subunit lead to distinct activation and inactivation recovery kinetics, significantly affecting Na_V_ channel regulation of cell excitability.

## Abstract (precis)

Voltage-gated Na_V_ channels are modulated by two different noncovalent accessory subunits: β1 and β3. Zhu et al. present data showing that β1 and β3 cause distinct effects on channel gating because they interact with Na_V_ channels at different locations. β3 regulates the voltage sensor in domain III, whereas β1 regulates the one in domain IV.

## Introduction

In electrically excitable organs, such as the heart, brain, and skeletal muscle, voltage-gated Na^+^ (Na_V_) channels cause the initiation and propagation of action potentials by conducting a large and rapid inward Na^+^ flux. Within the cells of these tissues, Na_V_ channels form macromolecular signaling complexes (Abriel, 2010) whose parts work in concert to regulate channel function. The Na_V_ β subunit members of this complex have been shown to regulate cell adhesion (Isom et al., 1995; Malhotra et al., 2000; Yu et al., 2003) and signaling in addition to affecting channel density (Calhoun and Isom, 2014), gating kinetics (Fahmi et al., 2001; Watanabe et al., 2009; Calhoun and Isom, 2014), and pharmacology (Lenkowski et al., 2003; Uebachs et al., 2010). However, the mechanisms whereby the β subunits interact with the Na_V_ channel α subunit to exert their influence on gating remain undiscovered.

Five types of Na_V_ β subunits have been identified: β1, β2, β3, β4, and β1b (Hartshorne and Catterall, 1984; Messner and Catterall, 1985; Kazen-Gillespie et al., 2000; Morgan et al., 2000; Yu et al., 2003). β2 and β4 form covalent disulfide bonds with the α subunit (Isom et al., 1995; Yu et al., 2003), whereas β1 and β3 interact non-covalently (Isom et al., 1992; Morgan et al., 2000). With the exception of the β1b splice variant (Patino et al., 2011), the β subunits comprise a single transmembrane domain that is tethered to an extracellular Ig loop and a cytoplasmic C terminus (Calhoun and Isom, 2014). Very recently, the covalently bound β2 and β4 subunits were crystallized (Gilchrist et al., 2013; Das et al., 2016), and a crucial disulfide bond formed by ^55^Cys in β2 and ^910^Cys in the DII pore loop was identified (Das et al., 2016). However, ^910^Cys is not present in Na_V_1.5, and instead the homologous position is ^868^Leu.

The β subunits are widely expressed in many tissues, including the central and peripheral nervous system, the heart, and skeletal muscle (Calhoun and Isom, 2014). Despite the sequence homology between non-covalently associated β1 and β3 subunits, their expression profile across organs differs. For instance, β1, but not β3, is highly expressed in skeletal muscles (The Human Protein Atlas). Intriguingly, even in the same organ, β subunit localization can differ (Fahmi et al., 2001; Calhoun and Isom, 2014; Yuan et al., 2014). For example, the β1 and β3 subunits have been shown to differentially express in the atria and ventricles (Fahmi et al., 2001; Watanabe et al., 2009; Yuan et al., 2014), suggesting that they may specifically tailor Na_V_ channel function according to cell type. Moreover, β1 and β3 also have a varied temporal expression profile during heart development. β1 expression has been shown to increase (Domínguez et al., 2005), whereas β3 has been shown to decrease through embryonic development (Okata et al., 2016). The dynamic expression patterns of β1 and β3 suggest that these two subunits play distinct roles in the regulation of Na_V_ channel function and the action potential.

The pore-forming Na_V_ channel α subunit is composed of four homologous domains (DI–DIV) connected by cytoplasmic linkers (Gellens et al., 1992). Each domain is formed by six α helical transmembrane segments (S1–S6). The fourth segments (S4) contain multiple positively charged residues that move across the membrane in response to changes in membrane potential. S4, together with S1–S3, form the voltage-sensing domains (VSDs) and are coupled to the S5 and S6, which form the channel pore. Upon membrane depolarization, the S4 segments within the VSDs of DI–DIII are propelled outward to open the channel within a millisecond; this is known as channel activation (Chanda and Bezanilla, 2002). Shortly thereafter, channels rapidly close—a process termed “fast inactivation” that is mediated by the intracellular DIII–DIV linker and the DIV-VSD (West et al., 1992). Both activation and inactivation gating have been previously shown to be modulated by the β1 and β3 subunits (Morgan et al., 2000; Fahmi et al., 2001; Watanabe et al., 2009; Calhoun and Isom, 2014).

Much mechanistic insight into Na_V_ channel gating has been recently provided by applying the voltage clamp fluorometry (VCF) protocol, which is used to fluorescently track VSD conformation and correlate VSD kinetics with ionic current gating. For many years, this protocol has been applied to study the skeletal muscle isoform Na_V_1.4, and it has provided great insight into the VSD roles in determining activation and inactivation gating kinetics (Cha et al., 1999; Chanda and Bezanilla, 2002; Silva and Goldstein, 2013a,b), the mechanisms of local anesthetic regulation of the VSDs (Muroi and Chanda, 2009; Arcisio-Miranda et al., 2010), and details of how toxins pathologically affect VSD activation (Campos et al., 2007, 2008). We have recently broadened this approach by developing VCF constructs to track VSD conformations of all four domains in the cardiac paralog, Na_V_1.5 (Varga et al., 2015; Zhu et al., 2016), whose ionic current modulation by the β subunit in oocytes mirrors the mammalian cell phenotype.

We hypothesized that the non-covalently bound β1 and β3 subunits would modulate Na_V_1.5 ionic current kinetics by altering the activation of one or more VSDs. In this study, we test this hypothesis by applying VCF to observe the β subunit effects on the VSD of each Na_V_1.5 domain.

## Materials and methods

### Molecular biology

cDNA encoding the human Na_V_ β3 (UniProtKB/Swiss-Prot under accession no. Q9NY72) subunit was custom synthesized by Life Technologies and inserted into the pBSTA plasmid. cRNAs for the human β1 subunit (UniProtKB/Swiss-Prot under accession no. Q07699.1) and α subunit Na_V_1.5 (accession no. Q14524.1) were produced from the pBSTA and pMAX vectors, respectively. All mutagenesis was accomplished using the QuikChange II site-directed mutagenesis kit (Agilent), with primers from Sigma-Aldrich. Multiple colonies were picked, and plasmids were isolated using the NucleoSpin plasmid miniprep kit (Macherey-Nagel). After samples were confirmed with sequencing (Genewiz), a single clone was selected for a Midiprep preparation (NucleoBond Xtra Midi; Macherey-Nagel). Each plasmid was then linearized with the NotI or EcoRI restriction enzyme and purified with the NucleoSpin Gel and PCR Clean-up kit (Macherey-Nagel). Finally, capped mRNA was synthesized in vitro using the mMESSAGE mMACHINE T7 Transcription Kit (Life Technologies), purified via phenol–chloroform extraction, and reconstituted to a concentration of ∼1 µg/µl.

### Cut-open oocyte recording

mRNAs for the human α subunit Na_V_1.5 and β1 or β3 subunits were injected at a 3:1 molar ratio (50–56 ng per cell total) into *Xenopus* oocytes. Oocytes were then incubated at 18°C in ND93 solution (93 NaCl mM, 5 KCl mM, 1.8 CaCl_2_ mM, 1 MgCl_2_ mM, 5 HEPES mM, 2.5 Na pyruvate mM, and 1% penicillin–streptomycin, pH 7.4). 3–7 d after injection, cut-open recordings (Stefani and Bezanilla, 1998; Rudokas et al., 2014) were performed using a cut-open amplifier (CA-1B; Dagan Corporation) coupled to an A/D converter (Digidata 1440; Molecular Devices). Clampex software (v10; Molecular Devices) was used for data acquisition. During recording, the temperature was maintained at 19°C with a controller (HCC-100A; Dagan Corporation). The internal recording solution was composed of 105 NMG-Mes mM, 10 Na-Mes mM, 20 HEPES mM, and 2 EGTA mM, at a pH level of 7.4, and the external solution was composed of 25 NMG-Mes mM, 90 Na-Mes mM, 20 HEPES mM, and 2 Ca-Mes_2_ mM, at a pH level of 7.4.

Before recording, the membrane capacitance compensation and P/–8 leak subtraction were applied. The ionic currents were recorded using the standard I-V protocol. From a holding potential of −120 mV, cells were stepped to a 100-ms prepulse of −120 mV and then stepped to test potentials ranging from −120 to 60 mV with a 10-mV increment, preceded by a 100-ms postpulse of −120 mV. For steady-state inactivation (SSI), cells were held at test potential for 200 ms; availability was then tested using a depolarizing pulse of −20 mV. Gating currents were recorded during test pulses from −150 to 50 mV from a holding potential of −120 mV. Capacitance and leak were compensated by P/4 leak subtraction with a subsweep potential of 40 mV. Gating charge–voltage (Q-V) curves were constructed by integrating gating currents over 7 ms after the voltage step.

### Voltage clamp fluorometry

Before recording, oocytes were labeled with 10 µmol/L methanethiosulfonate-carboxytetramethylrhodamine (MTS-TAMRA; Santa Cruz Biotechnology) in a depolarizing solution (in mM: 110 KCl, 1.5 MgCl_2_, 0.8 CaCl_2_, 0.2 EDTA, and 10 HEPES, pH 7.1) for 30 min on ice. Fluorescence data were collected simultaneously with ionic current on a custom rig (Varga et al., 2015), combining the cut-open voltage clamp and an epifluorescence upright microscope (FN1; Nikon), using a 40× water-immersion objective with 0.8 NA (CFI Plan Fluor; Nikon). A green, high-powered LED (Luminus; PT-121) was used for illumination, controlled by a driver (Lumina Power; LDPC-30-6-24VDC) by Clampex software. The emission light was measured with a photodiode (PIN-040A; United Detector Technology) mounted on the microscope epifluorescence port. The photocurrents generated by the photodiode were then amplified by a patch clamp amplifier (Axopatch-200A; Molecular Devices). Each fluorescence trace is a mean of 7–10 fluorescence recordings of the same cell.

### Data analyses

Data analyses were performed using Clampfit (v10; Molecular Devices), MATLAB (R2012a; MATLAB), and Excel (Microsoft). For fluorescence data, signals were low-pass filtered at 1 kHz offline before analysis. To correct for photobleaching, the baseline fluorescence trace, which has no change in voltage, was fit and subtracted from the traces recorded when the voltage protocol was applied.

Steady-state voltage dependence curves (G-V, fluorescence against voltage [F-V], SSI) were quantified by fitting a Boltzmann function: y = 1/(1 + exp[(V − V_1/2_)/k]). Sample sizes were chosen so that the standard error of mean was less than 0.1 for each data point, and a minimum sample size of three was determined to calculate the SD. Each data point shown reflects *n* = 3 or more from two or more batches of oocytes. Statistics for comparison between different constructs were performed using an independent *t* test (Microsoft Excel). The ± symbols in the text and table and the error bars in the figures represent the SEMs.

### Online supplemental material

The supplemental material contains data of channel currents properties that are not depicted in the main figures and other control data. Figs. S1 and S2 show channel activation and inactivation with or without β1 and β3 for Na_V_1.5 expressed in HEK 293T cells and for four VCF constructs. Fig. S3 shows the voltage dependence of fluorescence and gating charges for the decoupling mutations, A1330W and N1759A. Fig. S4 shows the fluorescence data from all four domains with S156W β1 or S155W β3. Fig. S5 shows DIII and DIV fluorescence deactivation kinetics comparisons for Na_V_1.5 expressed with β1/β3 chimeras.

## Results

### β1 regulates channel inactivation by altering voltage-dependent DIV-VSD transitions

**Figure 1.**
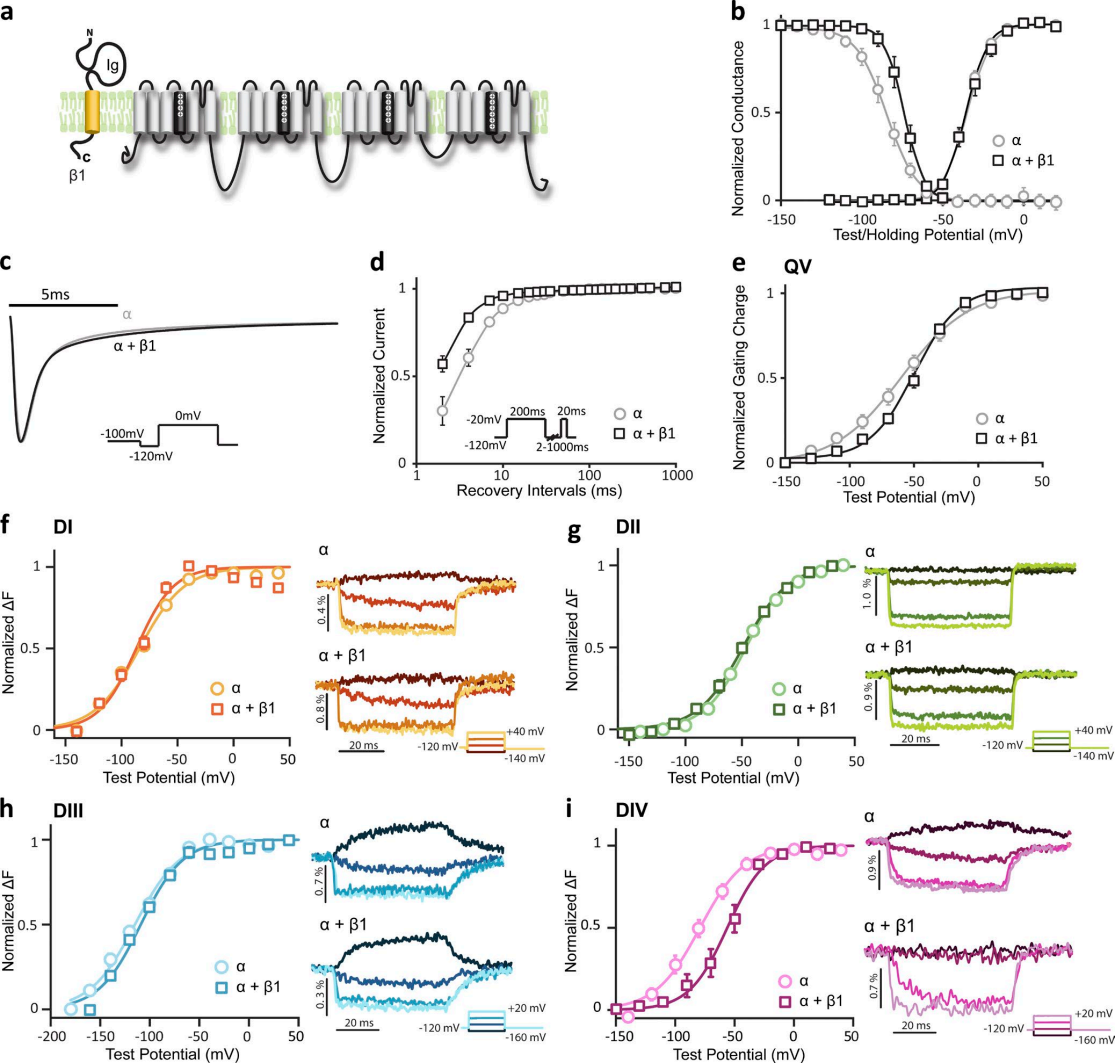
**Na_V_ β1 regulates Na_V_1.5 inactivation by altering DIV-VSD activation.** Na_V_1.5 ionic currents were measured using the cut-open voltage clamp technique to resolve fast Na^+^ channel kinetics. Changes in site-specific fluorescence of Na_V_1.5 are reported by four VCF constructs (V215C, S805C, M1296C, and S1618C) after conjugating to MTS-TAMRA (Varga et al., 2015). The mean ± SEM is reported for groups of three to eight cells. The error bars represent the SEMs. Some error bars are not visible due to small SEMs. (a) Topology of Na_V_1.5 and Na_V_ β1 subunits on plasma membrane. The β1 subunit is a single transmembrane protein containing an extracellular Ig domain and a short intracellular C terminus. (b) Voltage dependence of activation (G-V) and steady-state inactivation (SSI) for WT Na_V_1.5 with β1 (α + β1, square) or without β1 (α, circle). The G-V curve is constructed by measuring the peak current during test pulses from −120 to 20 mV from a holding potential of −120 mV and then dividing by the driving force (test pulse potential minus reversal potential). The reversal potential for each cell is determined individually. For the SSI curve, cells are held from −150 to 20 mV with a 10-mV increment for 200 ms. Availability is then measured by the peak current that results from a test pulse of −20 mV. Data are fit with a Boltzmann equation (solid lines), and parameters are reported in Table 1. (c) Representative current traces of WT channel with β1 (black) or without β1 (gray) in response to the depolarizing pulse to 0 mV from −120 mV. Current traces shown are constructed as a mean of three normalized current traces. Channels with or without β1 show comparable activation and inactivation kinetics. (d) Time dependence of fraction of current recovered for channels with β1 (α + β1, square) or without β1 (α, circle). Cells were first depolarized to −20 mV for 200 ms to induce inactivation; then, after various recovery durations at −120 mV, cells were depolarized to −20 mV to test the fraction of current recovered. (e) Gating charge–voltage (Q-V) curves of the WT LFS channel with β1 (α + β1, square) or without β1 (α, circle). Gating currents were recorded during test pulses from −150 to 50 mV from a holding potential of −120 mV. Capacitance and leak were compensated by P/4 leak subtraction with a subsweep potential of 40 mV. Q-V curves were constructed by integrating gating currents over 7 ms after the voltage step. (f–i, left) Voltage dependence of steady-state fluorescence (F-V curve) from all four domains—(f) DI-S216C, (g) DII-S805C, (h) DIII-M1296C, and (i) DIV-S1618C—coexpressed with β1 (α + β1, squares) or without β1 (α, circles). F-V curves are measured with 50-ms depolarizing pulses, ranging from −180 to 20 mV, with a 20-mV increment. The fluorescence change at each potential, ΔF, is determined by taking the mean of the signal amplitude after it reaches steady state. β1 coexpression causes a depolarizing shift in the DIV F-V curve without significantly affecting other domains. (f–i, right) Representative fluorescence signals showing the kinetics of VSD activation from each domain resulting from 50-ms depolarizing pulses ranging from −160 to 40 mV with 20-mV increments after a prepulse of −120 mV. For clarity, only four traces are shown for each construct. Percentage of fluorescence change (ΔF/F) is reported.

We coexpressed the human β1 subunit with the pore-forming Na_V_1.5 α subunit in *Xenopus* oocytes by coinjecting β1 and α subunit mRNA at a molar ratio of 3:1. β1 coexpression had no significant effect on the voltage dependence of WT channel activation, as shown by the conductance-voltage (G-V) curve (Fig. 1 b and Table 1) but caused a depolarizing shift in the channel steady-state inactivation (SSI) curve compared with WT alone (ΔV_1/2_ = 12.2 ± 1.4 mV, P = 0.02; Fig. 1 b). The right-shifted SSI curve implies that more channels are available to open at higher potentials. Moreover, β1 further increased channel opening by accelerating channel recovery from inactivation (Fig. 1 d). To ensure that the changes in the β1 regulation mechanism that we observed were consistent across different expression systems, we also used identical protocols to assess β1 effects on Na_V_1.5 currents in HEK 293T cells and observed similar behavior (Fig. S1 a). The β1-induced depolarization of SSI we observed is also consistent with previous results in HEK 293 and HEK 293T cells (An et al., 1998; Malhotra et al., 2001; Maltsev et al., 2009).

We investigated how β1 modulates inactivation by first measuring gating currents, which reflect charge translocation of all four VSDs. To be able to measure the gating current for the Na_V_1.5 channel, we used the WT LFS construct, which contains C373Y mutation that increases channel sensitivity to TTX, and the Y1977A mutation, which prevents ubiquitination of the channels to increase expression (Varga et al., 2015). Comparison between the gating charge–voltage dependence (Q-V) of WT LFS channels coexpressed with and without the β1 subunit (Fig. 1 e) revealed that β1 caused a depolarizing shift in the Q-V curve at negative potentials, resulting in a steeper Q-V relationship (Δk = −10.1 ± 4.2 mV, P = 0.04). This result suggests that in the presence of the β1 subunit, one or more of the VSDs requires higher potentials to activate. To identify which VSD was affected, Na_V_1.5 VCF constructs were coexpressed with the β1 subunit. We have previously shown that MTS-TAMRA–labeled Na_V_1.5 channels activate and inactivate similarly to WT channels (Varga et al., 2015). Coexpression of the β1 subunit with the VCF constructs caused a shift in the SSI curves that is similar to the shift caused by β1 in WT channels (Fig. S1 b). The voltage dependence of activation of each VSD can be described by plotting the steady-state fluorescence against voltage (F-V) curve. In comparison to α alone, the β1 subunit did not significantly alter the DI, DII, or DIII F-V curves (Fig. 1, f–h), but induced a strong depolarizing shift in the DIV F-V curve (ΔV_1/2_ = 31.3 ± 1.7 mV, P = 0.02; Fig. 1 i). Thus, in the presence of the β1 subunit, the DIV-VSD requires higher potentials to accomplish its activation transition, consistent with the gating charge shift (Fig. 1 e).

Previously, DIV-VSD activation was shown to be more closely linked to Na_V_ channel inactivation than activation (Cha et al., 1999; Capes et al., 2013). Specifically, the DIV-VSD was observed to be immobilized by fast inactivation (Cha et al., 1999), and DIV-VSD activation was shown to be the rate-limiting step for fast inactivation (Capes et al., 2013). Hence, changes in the voltage dependence of DIV-VSD activation or DIV-VSD kinetics would be expected to cause correlated changes in channel SSI or inactivation kinetics. Notably, DIV-VSD deactivation kinetics are also faster with β1 (t_100–10%_ = 4.5 ± 0.6 ms at −160 mV after 0-mV pulse) compared with α alone (t_100–10%_ = 13.2 ± 0.4 ms at −160 mV after 0-mV pulse, P = 0.0003). Thus, our results imply that the β1 subunit regulates inactivation by altering DIV-VSD transitions. This finding is consistent with the results of previous studies suggesting that β1 binds to the C terminus of Na_V_1.1 (Spampanato et al., 2004) and the S5–S6 linker of DIV of Na_V_1.4 (Makita et al., 1996). Our results build on these previous findings by connecting VSD regulation to altered inactivation kinetics.

Even though β1 does not affect the voltage dependence of DIII-VSD activation, comparison of DIII-VSD deactivation kinetics in the presence of β1 shows that DIII-VSD recovery to the resting position upon repolarization is faster and more complete (t_100–10%_ = 16.5 ± 1.6 ms at 0 mV) in contrast to α alone (t_100–10%_ = 23.9 ± 2.3 ms at 0 mV, P = 0.05; Fig. 1 h, right). In previous studies, fast inactivation was shown to immobilize the gating charge displaced by DIII and DIV (Armstrong and Bezanilla, 1977; Cha et al., 1999), particularly DIII-VSD (Sheets and Hanck, 2005; Varga et al., 2015). Our results suggest that β1 allows the DIII and DIV VSDs to recover to the resting state more quickly. Given the link to inactivation, this more rapid recovery of the VSDs will allow channels to recover more quickly from inactivation (Fig. 1 d) and become available for excitation in a shorter amount of time.

### β3 alters channel activation and inactivation by modulating DIII and DIV VSD kinetics

**Figure 2.**
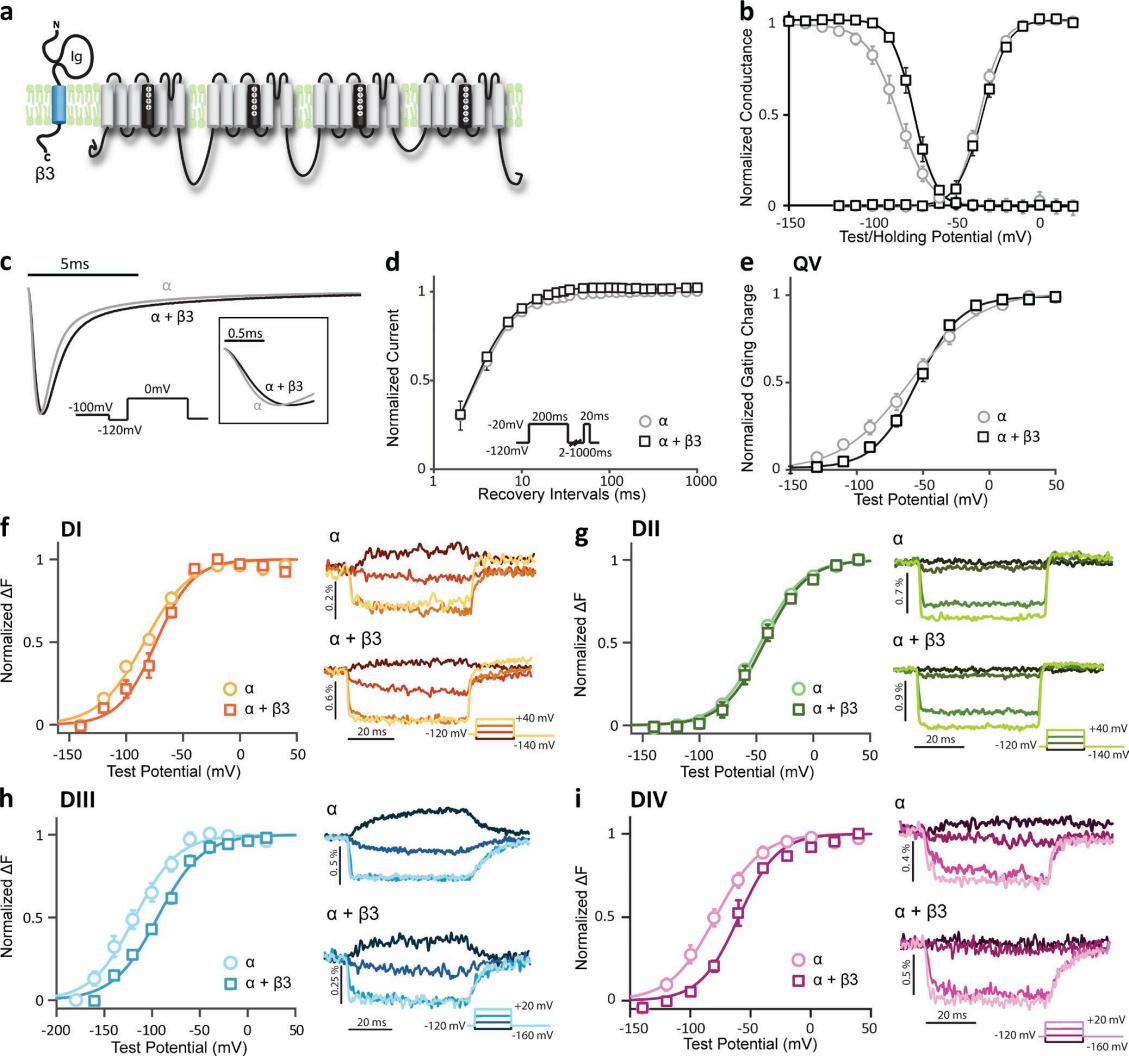
**Na_V_ β3 subunit affects Na_V_1.5 inactivation by modifying both DIII and DIV VSD activation.** Na_V_1.5 ionic currents and site-specific fluorescence changes are measured as described in Fig. 1. Groups of 3–10 cells are reported as mean ± SEM. (a) Topology of Na_V_1.5 and Na_V_ β3 subunits on plasma membrane. The Na_V_ β3 subunit structure is homologous to the β1 subunit, and it has also been shown to express in the myocardium (Hu et al., 2012). (b) Voltage dependence of activation (G-V) and steady-state inactivation (SSI) for WT Na_V_1.5 with β3 (α + β3, squares) or without β3 (α, circles). The G-V curve and SSI curve were constructed and recorded as shown in Fig. 1. Boltzmann fit parameters are listed in Table 1. (c) Representative current traces of WT channel with β3 (black) or without β3 (gray) in response to depolarizing the pulse to 0 from −120 mV. Channels with β3 show slower activation and deactivation kinetics compared with α alone. (d) Time dependence of fraction of current recovered for channels with β3 (α + β3, black squares) or without β3 (α, gray circles). The same protocol was used as shown in Fig. 1. (e) Gating charge–voltage (QV) curve for WT LFS Na_V_1.5 with β3 (α + β3, squares) or without β3 (α, circles). The Q-V curve was recorded and constructed as shown in Fig. 1. Boltzmann fit parameters are listed in Table 1. (f–i, left) Voltage dependence of fluorescence (F-V curve) from four VCF constructs—(f) DI-V215C, (g) DII-S805C, (h) DIII-M1296C, and (i) DIV-S1618C—coexpressed with β3 (α + β3, squares) or without β3 (α, circles). F-V curves are constructed and recorded as shown in Fig. 1. β3 coexpression causes depolarizing shifts in DIII and DIV F-V without significantly affecting the other two domains. (f–i, right) Representative fluorescence signals representing the kinetics of each VSD activation. For clarity, only four traces with a 40-mV interval are shown for each construct.

We coexpressed the β3 subunit with Na_V_1.5 using the same protocols that were used for β1 (Fig. 2 a). As with the β1 subunit, β3 had no apparent effect on the voltage dependence of channel activation (G-V; Fig. 2 b), but slowed ionic current activation and inactivation kinetics (Fig. 2 c). It also caused a depolarizing shift (ΔV_1/2_ = 8.7 ± 1.5 mV, P = 0.02) in SSI (Fig. 2 b and Table 1), implying that β3 expression increases Na_V_1.5 channel availability at higher potentials. A similar β3-induced SSI shift was also present in HEK cells recorded with identical protocols (Fig. S2 a). Unlike β1, β3 does not significantly alter channel recovery kinetics (Fig. 2 d). When we coexpressed β3 with the four VCF constructs, the gating effects of β3 were preserved (Fig. S2 b), except with the DII LFS construct, where the shift in SSI induced by β3 is less pronounced. Our observations of the ionic current changes induced by β3 coexpression are consistent with the gating effects shown previously in oocytes (Fahmi et al., 2001) and the *scn3b* knockout mouse phenotype (Hakim et al., 2008).

**Table 1.** Parameters of Boltzmann fit to G-V, SSI, and F-V curves for WT Na_V_1.5 or VCF constructs expressed with or without WT β1 or β3

**Parameter**	**DI**	**DI + β1**	**DI + β3**	**DII**	**DII + β1**	**DII + β3**
**G-V**						
V_1/2_	−42.3 ± 1.7	−50.2 ± 1.0	−37.1 ± 1.8	−33.9 ± 2.2	−38.9 ± 3.0	−43.5 ± 2.2
k [n]	8.16 ± 0.7 [5]	8.3 ± 0.6 [4]	9.7 ± 0.5 [4]	9.5 ± 0.2 [4]	8.5 ± 1.2 [9]	−7.0 ± 0.5 [4]
**SSI**						
V_1/2_	−96.3 ± 4.55	−78.6 ± 2.1	−79.6 ± 2.4	−88.8 ± 1.3	−79.2 ± 4.5	−86.4 ± 0.7
k [n]	−11.0 ± 1.1 [4]	−6.4 ± 0.2 [8]	−7.0 ± 0.4 [4]	−8.2 ± 0.4 [4]	−6.5 ± 0.9 [7]	−6.1 ± 0.1 [4]
**F-V**						
V_1/2_	−111.5 ± 1.0	−92.1 ± 11.4	−75.7 ± 4.6	−48.4 ± 2.7	−51.1 ± 3.5	−45.5 ± 5.0
k [n]	21.3 ± 2.4 [4]	18.6 ± 2.3 [4]	15.3 ± 2.5 [4]	19.4 ± 0.7 [4]	23.1 ± 2.2 [6]	19.3 ± 0.8 [4]
	**DIII**	**DIII + β1**	**DIII + β3**	**DIV**	**DIV + β1**	**DIV + β3**
**G-V**						
V_1/2_	−43.7 ± 1.9	−40.0 ± 4.4	−39.2 ± 1.4	−36.8 ± 1.6	−34.6 ± 3.4	−38.2 ± 1.3
k [n]	7.8 ± 0.6 [5]	9.4 ± 0.7 [13]	7.4 ± 0.5 [6]	8.9 ± 0.9 [4]	9.2 ± 0.6 [19]	7.2 ± 0.5 [5]
**SSI**						
V_1/2_	−94.7 ± 1.9	−76.2 ± 2.5	−86.0 ± 1.6	−91.7 ± 3.4	−74.2 ± 3.1	−78.0 ± 1.6
k [n]	−9.8 ± 0.7 [4]	−6.7 ± 0.4 [7]	−7.6 ± 0.4 [4]	−12.6 ± 0.9 [5]	−10.3 ± 0.9 [13]	−9.2 ± 0.5 [5]
**F-V**						
V_1/2_	−120.7 ± 4.8	−122.1 ± 1.4	−98.0 ± 2.6	−88.2 ± 5.3	−56.8 ± 6.6	−63.2 ± 4
k [n]	25.6 ± 0.7 [4]	24.3 ± 1.3 [5]	26.4 ± 2.0 [5]	23.6 ± 3.1 [4]	14.5 ± 2.5 [6]	14.4 ± 0.3 [4]
	**WT α**	**WT α + β1**	**WT α + β3**			
**G-V**						
V_1/2_	−35.8 ± 1.4	−34.6 ± 1.9	−30.4 ± 1.9			
k [n]	6.8 ± 0.2 [4]	7.2 ± 0.7 [5]	7.4 ± 0.3 [3]			
**SSI**						
V_1/2_	−84.8 ± 2.5	−74.0 ± 2.4	−75.2 ± 1.9			
k [n]	−8.9 ± 0.7 [5]	−5.7 ± 0.3 [4]	−6.2 ± 0.3 [3]			
	**WT LFS α**	**WT LFS α + β1**	**WT LFS α + β3**			
**Q-V**						
V_1/2_	−60.7 ± 6.9	−49.8 ± 3.1	−55.1 ± 2.3			
k [n]	25.9 ± 2.8 [4]	17.3 ± 1.2 [3]	17.3 ± 2.5 [8]			

Like β1, β3 caused a depolarizing shift in the Q-V curve at negative potentials and a steeper Q-V relationship (Δk = 8.6 ± 3.0 mV, P = 0.05), showing that β3 also alters the voltage dependence of VSD activation. Considering the homology between the β1 and β3 subunits, the similar Q-V curves are not surprising. However, comparison of the F-V curves of α alone and α with β3 shows that β3 induces a depolarizing shift in the DIII F-V curve (DIII F-V: ΔV_1/2_ = 20.7 ± 3.9 mV, P = 0.01) in addition to its depolarizing effect on the DIV F-V curve (DIV F-V: ΔV_1/2_ = 25.0 ± 7.7 mV, P = 0.01; Fig. 2, h and i; and Table 1), causing both the DIII and DIV VSDs to activate at higher potentials. The DIV F-V depolarizing shift occurs over the same potential range as the shift in SSI, consistent with the findings that DIV-VSD activation strongly correlates with channel inactivation and with the aforementioned experiments with β1.

Channel opening is known to be regulated by DIII-VSD activation (Muroi et al., 2010; Wang et al., 2016). Yet, β3 induced depolarization of DIII-VSD activation without affecting the channel voltage dependence of activation (G-V), which may be due to DIII-VSD activation at very negative potentials in the Na_V_1.5 paralog. Still, β3 slowed ionic current activation kinetics (α alone: dI/dt_max_ = 1.6 ± 0.1 ms^−1^; α + β3: dI/dt_max_ = 1.1 ± 0.1 ms^−1^, P = 0.04; Fig. 2 c). Slower inactivation rates can also result in slower activation kinetics when normalized currents are compared because channel activation and inactivation are tightly coupled (Aldrich et al., 1983). Thus, the slowed activation kinetics we observed with β3 could alternatively be caused by slowed inactivation kinetics. In contrast to β1, β3 only accelerates DIII-VSD deactivation (α + β3: t_100–10%_ = 9.2 ± 1.3 ms; α alone: t_100–10%_ = 23.9 ± 2.3 ms, P = 0.005), but not DIV-VSD deactivation (see Table 4). In the Na_V_1.5 channel, the DIII-VSD activates at very negative potentials (∼160 mV). Thus, it is a technical challenge to acquire the negative baseline of the DIII F-V curve for the more hyperpolarized shifted constructs (α alone and α + β1). Despite this limitation, we expect that the hyperpolarized shifted constructs would have more negative V_1/2_ if we were able to record to the baseline, suggesting that the DIII depolarizing shift induced by β3 is even larger than reported.

Despite being highly homologous to β1, β3 is unique in altering the VSD transitions of both DIII and DIV. The DIV-VSD effects induced by β3 are similar to those induced by β1, causing a depolarizing SSI shift. The β3 effect on the DIII-VSD, which shifts DIII-VSD’s activation to higher potentials, slows ionic current activation and inactivation kinetics. Two α–β3 interaction mechanisms could explain the changes in the VSD movements that we observed. One possibility is that β3 can interact with both DIII and DIV. A second plausible mechanism is that β3 mainly interacts with the DIII-VSD, which can allosterically modify the adjacent DIV-VSD activation. In the following sections, results from β1/β3 chimera and α–β3 quencher fluorophore pair experiments support the latter mechanism.

### High expression of β3 separates DIII VSD activation into two steps

To ensure that the VSD alterations we observed were truly caused by the expression of β subunits and that the amount of β subunits expressed on the membrane saturated the modulation effects of Na_V_1.5 channels, we tested different expression levels of β subunits. We altered β subunit expression levels by injecting mRNAs encoding α and β subunits at different molar ratios, observing their effects on ionic current and VSD activation.

**Figure 3.**
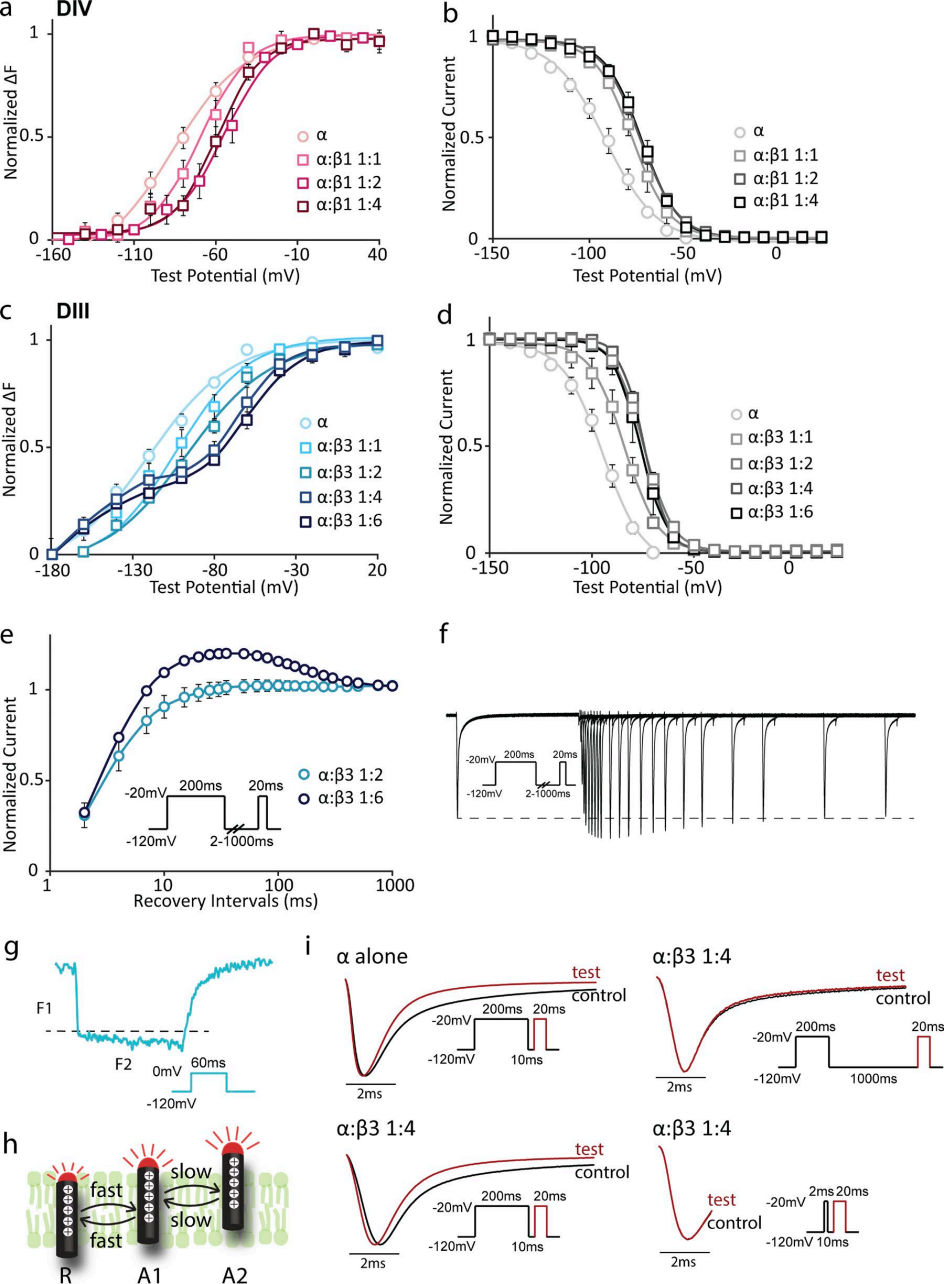
**High expression of β3 subunits separate DIII-VSD movements into two components.** Groups of three to five cells are reported as mean ± SEM. (a and b) The DIV VCF construct (α) was coinjected with β1 mRNA at molar ratios of 1:1, 1:2, and 1:4 or without β. (a) The DIV F-V curve was constructed for each molar ratio of α:β1. The β1-induced depolarizing shift of DIV F-V saturates when molar ratio of α:β1 reaches 1:2. (b) Channel steady-state inactivation (SSI) curves were constructed for the same molar ratios of α and β1. The depolarizing shift of the SSI curve caused by β1 saturates when the molar ratio of α:β1 reaches 1:2. (c and d) The DIII VCF construct (α) was coinjected with β3 mRNA at molar ratios of 1:1, 1:2, 1:4, and 1:6 or without β. (c) DIII F-V was constructed for each molar ratio of α:β3. When α:β3 is greater than 1:4, DIII F-V starts to exhibit two activation steps. (d) Channel SSI curves were constructed for the same molar ratios of α and β3. β3-induced SSI depolarization saturates at a 1:2 α:β3 molar ratio. (e) Comparison between channel recovery from inactivation curves for α:β3 at 1:2 and 1:6. To assess the time dependence of channel recovery from inactivation from a holding potential of −120 mV, channels were stepped and held at −20 mV for 200 ms, stepped back to −120 mV for a different amount of time (2–1,000 ms), and then stepped up to 20 mV to test availability. (f) Representative channel recovery from inactivation current traces at a 1:6 α:β3 molar ratio. The dotted line represents the first pulse peak amplitude. (g) DIII fluorescence trace at a 1:6 α:β3 molar ratio in response to 0 mV depolarizing potential. The fluorescence shown is a mean of traces from three cells. In parallel to DIII fluorescence voltage dependence, the DIII fluorescence activation kinetics also display two components: F1 and F2. (h) Schematic model of DIII VSD movements when channels are coassembled with high expression level of β3. (i) Comparison of the current activation kinetics shows that with high expression of the β3 subunit, the second current pulse after a short recovery time has faster activation kinetics. (i, top left) Comparing the first and second current traces evoked by depolarizing pulse at −20 mV with a 10-ms recovery time between these two pulses for channels expressed without the β subunit. The second pulse has the same rise time but faster inactivation. (i, bottom left) Channels were overexpressed with the β3 subunit at a 1:4 α:β3 molar ratio. Comparison of the first and second current traces evoked by depolarizing pulse at −20 mV with a 10-ms recovery time between these two pulses for channels overexpressed with the β3 subunit. The second current pulse has faster activation kinetics in contrast to α alone. (i, top right) Channels were overexpressed with the β3 subunit at a 1:4 α:β3 molar ratio. Comparison of the first and second current traces evoked by depolarizing pulse at −20 mV with a 1,000-ms recovery time in between. The first and second pulses have similar activation kinetics. (i, bottom right) For channels overexpressed with the β3 subunit at a 1:4 α:β3 molar ratio, the first voltage pulse was shortened to 2 ms. Comparing the first and second pulses after 10 ms of recovery, the activation kinetics remain the same.

For the β1 subunit, as we increased the mRNA molar ratio from 1:1 to 1:2, the DIV F-V curve shifted to more depolarized potentials (1:1 α:β1: V_1/2_ = −70.1 ± 5.2 mV, 1:2 α:β1: V_1/2_ = −56.8 ± 5.0 mV). Further, when the α:β1 mRNA molar ratio was increased to 1:4, the DIV F-V curve overlapped with the F-V curve of a 1:2 α:β1 mRNA molar ratio (Fig. 3 a), suggesting that β1 modulation of DIV-VSD saturated at a 1:2 α:β1 ratio. Consistently, the β1 alteration of channel SSI followed a similar saturation pattern (Fig. 3 b). This result further supports the idea that β1 regulates channel inactivation by altering DIV-VSD activation, an effect that saturates at a 1:2 ratio.

For β3, the saturation behavior was more complex. When we increased the mRNA molar ratio from 1:1 to 1:2, the DIII F-V curve shifted to depolarized potentials (1:1 α:β3: V_1/2_ = −108.6 ± 5.6 mV; 1:2 α:β3: V_1/2_ = −97.9 ± 2.6 mV; Fig. 3 c). Intriguingly, when the molar ratio of α:β3 was increased to 1:4 or higher, the DIII F-V curve started to exhibit two components that could no longer be fit with a single Boltzmann function (Fig. 3 c). The curve was well fit with two Boltzmann curves, one at very negative potentials (−180 to −80 mV), and the other within the channel activation voltage range (−80 to 20 mV). Correspondingly, the DIII fluorescence kinetics also followed two steps, which also were not well fit with a single exponential. The first rapid transition occurred within 2 ms after depolarization, denoted by F1, followed by a very slow component, denoted by F2, over a time period of 60 ms (Fig. 3 g).

The separation of two components in DIII-VSD activation caused unusual recovery from inactivation, where the peak current magnitude during the test pulse was larger than that during the control pulse after recovery times of 10 to 300 ms (Fig. 3, e and f). After 500- to 1,000-ms recovery at −120 mV, the peak current during the test pulse returned to the magnitude of the control pulse. Typically, Na^+^ currents exhibit monotonic behavior during this protocol.

We assessed the relationship between the two DIII-VSD activation components and channel recovery from inactivation by aligning current during the first control pulse with that of the second test pulse after 10- or 1,000-ms recovery (Fig. 3 i, bottom left, top right). For channels expressed without the β subunit, current during the test pulse after 10-ms recovery activated at the same rate as the control pulse (Fig. 3 i, top left). For channels coexpressed with the β3 subunit at a 1:4 molar ratio, current during the test pulse after 10-ms recovery activated more quickly in comparison with the control pulse (Fig. 3 i, bottom left). In contrast, the current during the test pulse after 1,000-ms recovery rose at the same rate as that of the control pulse (Fig. 3 i, top right). Faster channel activation kinetics can significantly increase peak current. High β3 expression causes faster activation kinetics during a test pulse that follows a short recovery interval (10–300 ms), resulting in peak current that exceeds the control pulse current.

To account for this behavior, we first suppose that the DIII-VSD activates in two steps from resting (R) to intermediate activated (A1) and activated (A2) states (Fig. 3 h). We then assume that the transition from R to A1 is fast and is described by the F1 component, whereas the transition from A1 to A2 is slow, as shown by the F2 component. In this model, pore opening is facilitated by the transition of the DIII-VSD to the A1 state and further encouraged by entry into the A2 state. During the first 200-ms pulse, most DIII-VSDs are brought to A2. Because the transition from A2 to A1 is slow, when channels were given 10–300 ms to recover at −120 mV, the time was too short for DIII VSD to recover to A1, resulting in most of the DIII-VSDs being trapped in the A2 state. As most of the DIII-VSDs were still in the A2 state and it greatly facilitates pore opening, channel activation was faster for the second pulse. If this scheme is correct, we would predict that if we only allow the DIII-VSD to enter A1 by applying a short 2-ms depolarizing pulse as the control pulse, the second pulse will not have faster rising kinetics compared with the control pulse. Indeed, comparison of the control and test pulses shows that both pulses completely overlap (Fig. 3 i, bottom right).

It is unlikely that the physiological assembly of α–β3 will reach a ratio high enough to separate DIII-VSD movement into two components (Yuan et al., 2014). It is possible that overexpression of β3 will force some of the β3 subunits into a secondary low-affinity binding site. Consequently, if β3 is locally expressed at very high levels, this group of cells will have a relatively short refractory period and become particularly excitable because of the unique channel recovery behavior.

### **β**1 and **β**3 do not regulate VSD activation from the Na_V_1.5 pore via the S4–S5 linkers

By probing the VSD dynamics, we discovered that β1 and β3 modulate channel current by altering DIII and DIV VSD activation. However, the mechanism by which the β1 and β3 subunits interact with the Na_V_ channel to alter VSD activation remains unclear. The recent cryo-electron microscopy (cryo-EM) structure of the eukaryotic Na_V_ channel shows that the pore loops of the Na_V_ channel have bulky extracellular structures, making them good candidates for β subunit binding (Shen et al., 2017). To test if the β1 and β3 subunits allosterically modulate the VSDs by interacting with the DIII and DIV pore domains, we assessed β1 and β3’s effects on mutant channels where DIII or DIV VSD is decoupled from the pore. The interaction between the S4–S5 linker and the S6 is known to be essential for canonical coupling between the VSD and the pore of each domain. We used mutations that have previously been found to disrupt this type of coupling.

**Figure 4.**
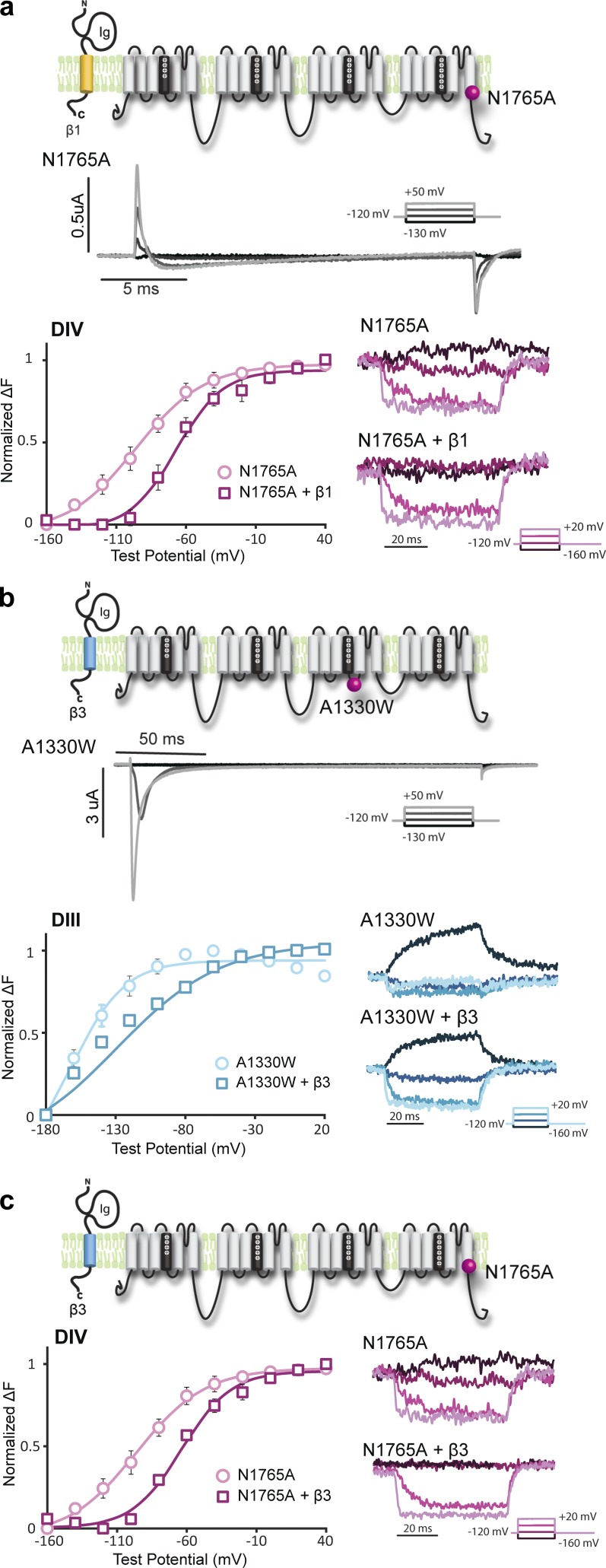
**β1 and β3 modulate the DIII and DIV VSDs, even when the VSDs are decoupled from the pore.** Groups of three to five cells are reported as mean ± SEM. (a) β1 coexpressed with the N1765A mutant channel. Representative ionic currents of the N1765A channel are shown. The N1765A channel has significantly reduced ionic currents but robust gating currents. Voltage dependence of fluorescence (F-V) of DIV-1618C for the N1765A channel expressed with β1 (N1765A + β1, squares) or without β1 (N1765A, circles). Parameters for Boltzmann fit are reported in Table 2. (b) β3 coexpressed with the A1330W mutant channel. Representative ionic currents of the A1330W channel show that the A1330W channel still conducts ionic currents. F-V of DIII-M1296C for the A1330W channel expressed with β3 (A1330W + β3, squares) or without β3 (A1330W, circles). As A1330W mutation hyperpolarizes the DIII F-V, the DIII F-V did not reach saturation even at −180 mV. This may result in an inaccurate measurement of the V_1/2_. However, from the representative fluorescence traces, it is very clear that β3 depolarizes the voltage dependence of DIII VSD activation. (c) β1 coexpressed with the N1765A mutant channel. F-V curves of DIV-S1618C for the N1765A channel with β3 (A1330W + β3, squares) or without β3 (A1330W, circles).

Residue N1765, which is on the S6 of DIV, has previously been shown to be essential for coupling the DIV-VSD to the pore (Sheets et al., 2015). Mutating N1765 to alanine (A) abolishes most of the ionic current (Fig. 4 a and Table 2) without affecting DIV-VSD movement (Fig. S3 a) and gating currents (Fig. S3 c), suggesting that the DIV-VSD is decoupled from the pore. Coexpressing β1 with the N1765A channel still depolarized the Q-V curve (Fig. S3 c) and the DIV F-V curve (Fig. 4 a), similar to the β1 effect on WT channels. If β1 interacts with the DIV pore domain to allosterically modulate the DIV-VSD through the S4–S5 linker, decoupling of the DIV-VSD from the DIV pore domain should abolish most of the β1 effect on the DIV-VSD. Instead, we found that the DIV-VSD of the N1765A is still modulated by β1, suggesting that β1 does not regulate the DIV-VSD via pore coupling through the DIV S4–S5 linker but instead interacts with the DIV-VSD directly or via an alternative pore–VSD coupling mechanism.

**Table 2.** Parameters of Boltzmann fit to the DIII and DIV F-V curves for N1765A and A1330W channels expressed with β1 or β3 subunits

**Parameter**	**A1330W alone**	**A1330W + β3**	**Parameter**	**N1765A alone**	**N1765A + β1**	**N1765A + β3**
**DIII F-V**			**DIV F-V**			
V_1/2_	−159.9 ± 4.9	−137.3 ± 5.1	V_1/2_	−99.3 ± 8.8	−66.7 ± 5.2	−62.6 ± 3.6
k [n]	18.7 ± 2.2 [3]	35.7 ± 0.8 [3]	k [n]	26.3 ± 4.4	16.9 ± 2.0	18.3 ± 2.4

Because β3 was shown to alter DIV-VSD activation (Fig. 2), we also tested the β3 effect on the N1765A channel. Like β1, β3 still causes depolarizing shifts in the DIV F-V curve (Fig. 4 c) and the voltage dependence of gating charges (Q-V; Fig. S3 d) of the N1765A channel, suggesting that β3 does not interact with the DIV pore domain to regulate DIV-VSD activation via the S4–S5 linker. In addition to the DIV effect, we also showed that the DIII-VSD is significantly affected by β3. We assessed the β3 effects on channels that contain a mutation that decouples the DIII pore from its VSD. A1149, located on the DIII S4–S5 linker of Na_V_1.4, is one of the key residues on the gating interface (Muroi et al., 2010). Mutating A1149 to W in the Na_V_1.4 stabilizes the activated DIII-VSD, but not the pore, suggesting that the coupling between the DIII-VSD and the DIII pore is reduced (Muroi et al., 2010). The homologous residue in the Na_V_1.5 isoform is A1330. Consistent with previous studies, A1330W greatly hyperpolarized the DIII F-V curve (Fig. S3 b) compared with WT. However, ionic current activation (G-V) was not significantly affected (Fig. S3 b). When coexpressed with β3, the DIII F-V curve was still significantly depolarized (ΔV_1/2_ = 22.6 ± 6.7 mV, P = 0.03) compared with A1130W alone (Fig. 4 b and Table 2). This result suggests that β3 does not regulate DIII-VSD activation via S4–S5 linker coupling to the DIII pore domain. However, there are alternate means to couple the pore to the VSD, including the DIII–DIV linker, and these alternative mechanisms could possibly be in play.

### Assessing β subunit localization

Because we observed that β1 and β3 modulate channel gating by affecting the DIII and DIV VSDs, we hypothesized that β1 and β3 are proximally located to these VSDs. To test this hypothesis, we introduced a fluorophore or a quenching tryptophan into the β subunit. This method was previously applied to other proteins, such as the T4 lysozyme (Mansoor et al., 2002, 2010) and the BK channel (Pantazis and Olcese, 2012), to map distances within proteins. We reasoned that if β1 or β3 resides near one of the channel’s VSDs, introducing a tryptophan residue to the top of the transmembrane segment of β1 or β3 would quench the fluorophore attached to the S3–S4 linker of that VSD.

We first introduced a tryptophan mutation to the extracellular region of the β1 transmembrane segment, S156W. Compared with WT β1, S156W β1 only slightly depolarizes the DI and DIV F-V curves, but none of the domains’ fluorescence signals were significantly quenched (Fig. S4). This result suggests that the S156 residue of β1 is not within detectable quenching distance to the S4s of any domain.

**Figure 5.**
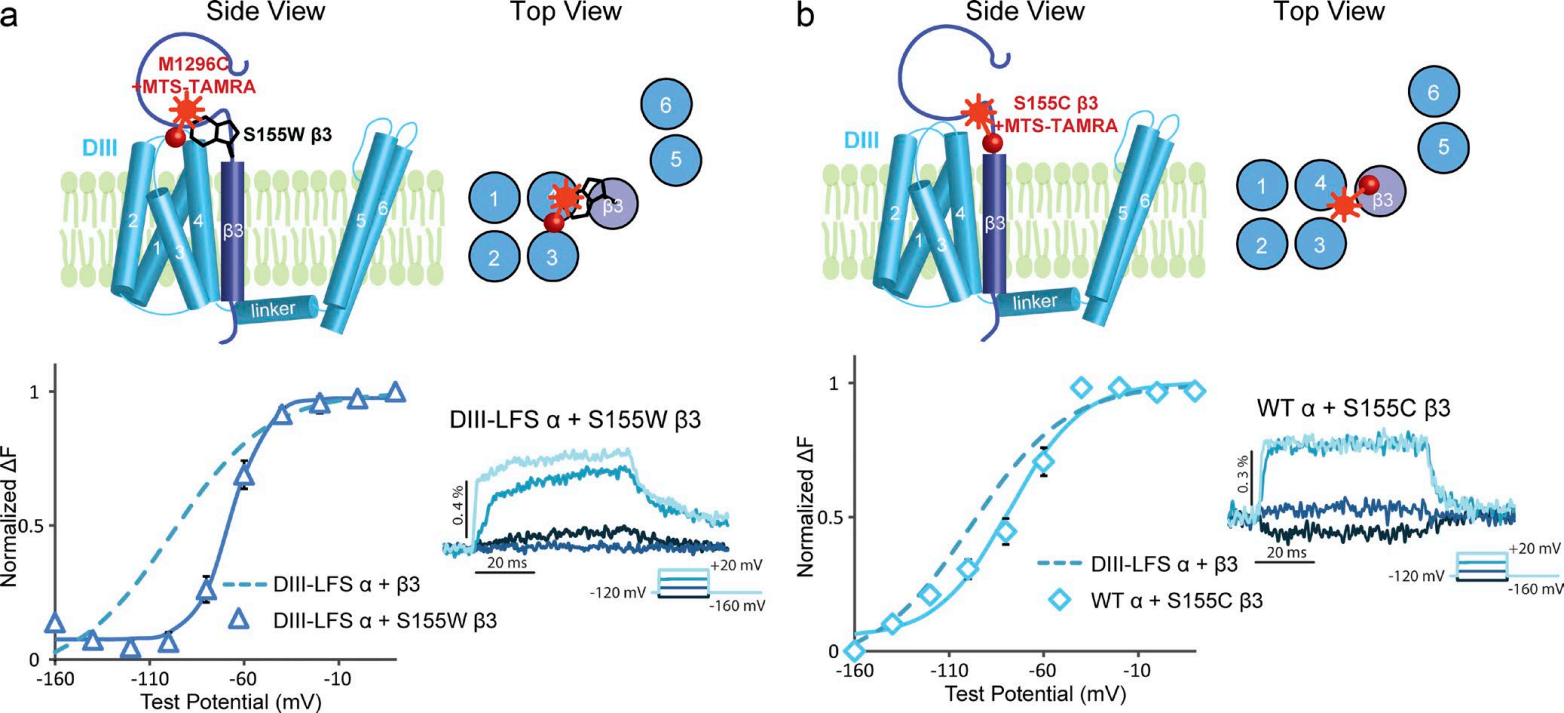
**Tryptophan-induced quenching of the fluorophore method reveals β3 proximity to the DIII VSD.** (a, top) Side view and top view showing the proposed location of the β3 subunit with respect to the channel. Only DIII is shown for clarity. A tryptophan mutation is made on the extracellular β3 domain. In this proposed assembly of α and β3, the tryptophan residue is very close to the DIII LFS labeling site M1296C. (a, bottom) Fluorescence-voltage (F-V) curve of DIII LFS coexpressed with S155W β3 (triangles) in comparison to the F-V curve of DIII LFS coexpressed with WT β3 (dotted line). Representative fluorescence traces from the DIII LFS in response to different voltages. The fluorescence from DIII LFS increases upon membrane depolarization. (b, top) Side view and top view showing the WT α subunit coexpressed with S155C β3 that is labeled with MTS-TAMRA. (b, bottom) F-V curve of WT α coexpressed with the labeled S155C β3 (diamonds) compared with the DIII LFS coexpressed with WT β3 (dotted line). The representative fluorescence traces from WT α coexpressed with the labeled S155C β3 are shown on the right.

We then introduced a tryptophan mutation into the extracellular region of the β3 transmembrane segment, S155W. S155W β3 did not affect DI, DII, or DIV F-V curves or fluorescence compared with WT β3, but the fluorescence of the DIII-VSD was completely reversed (Fig. 5 a). In the DIII LFS construct, the DIII-VSD fluorescence signal moves downward upon membrane depolarization (Fig. 2 h). This reduction in fluorescence is consistent with local environment quenching of the fluorophore attached to the S3–S4 linker of DIII upon activation. In contrast, when S155W β3 is present, its tryptophan strongly quenches the fluorophore on the DIII S3–S4 linker when S4 is at resting position. When S4 activates, the fluorophore moves away from the tryptophan, resulting in an increase in fluorescence. This result shows that the β3 subunit resides very close to the DIII-VSD at a distance that is within the van der Waals contact distance (5–15 Å; Mansoor et al., 2002). Notably, the DIII fluorescence kinetics with S155W β3 are greatly slowed, and the F-V curve has similar voltage dependence (V_1/2_ = −67.2 ± 3.5 mV) as the second component of DIII F-V with high β3 expression (Fig. 3 c). Thus, the DIII fluorescence with S155W β3 tracks a slower component of the DIII-VSD that occurs at higher potentials, a component that is observed but not prominent with the LFS construct.

If β3 and the DIII-VSD are close in proximity, we would also expect to track DIII-VSD conformational changes by labeling the β3 subunit. To test this hypothesis, we introduced a cysteine into the extracellular β3 segment, S155C. We coexpressed S155C β3 with the WT Na_V_1.5 α subunit in *Xenopus* oocytes. We then labeled the S155C β3 with a fluorophore (MTS-TAMRA). A voltage-dependent fluorescence signal was detected (Fig. 5 b). Changes in the local environment of the fluorophore attached to β3 when DIII-VSD changed conformation likely produced this signal. The F-V curve of the WT α subunit with the labeled S155C β3 subunit is comparable to the F-V curve of the labeled DIII LFS α subunit with the WT β3 subunit (Fig. 5 b). This result suggests that the fluorescence signal generated by the labeled β3 subunit represents the conformational changes of the DIII-VSD.

Using the tryptophan-induced fluorophore quenching method (Mansoor et al., 2002, 2010; Pantazis and Olcese, 2012), we demonstrated β3’s proximity to the DIII-VSD. Given that β3 still affects the DIII-VSD that is decoupled from the pore by the A1330W mutation and the location of the β3 subunit, we conclude that β3 modulates the DIII-VSD by direct interaction. Taking advantage of β3 proximity to the DIII-VSD, we are now able to track the DIII-VSD conformation without directly labeling the α subunit.

### β1 and β3 chimeras show that both the extracellular and transmembrane domains of β3 are essential for its interaction with the DIII-VSD

**Figure 6.**
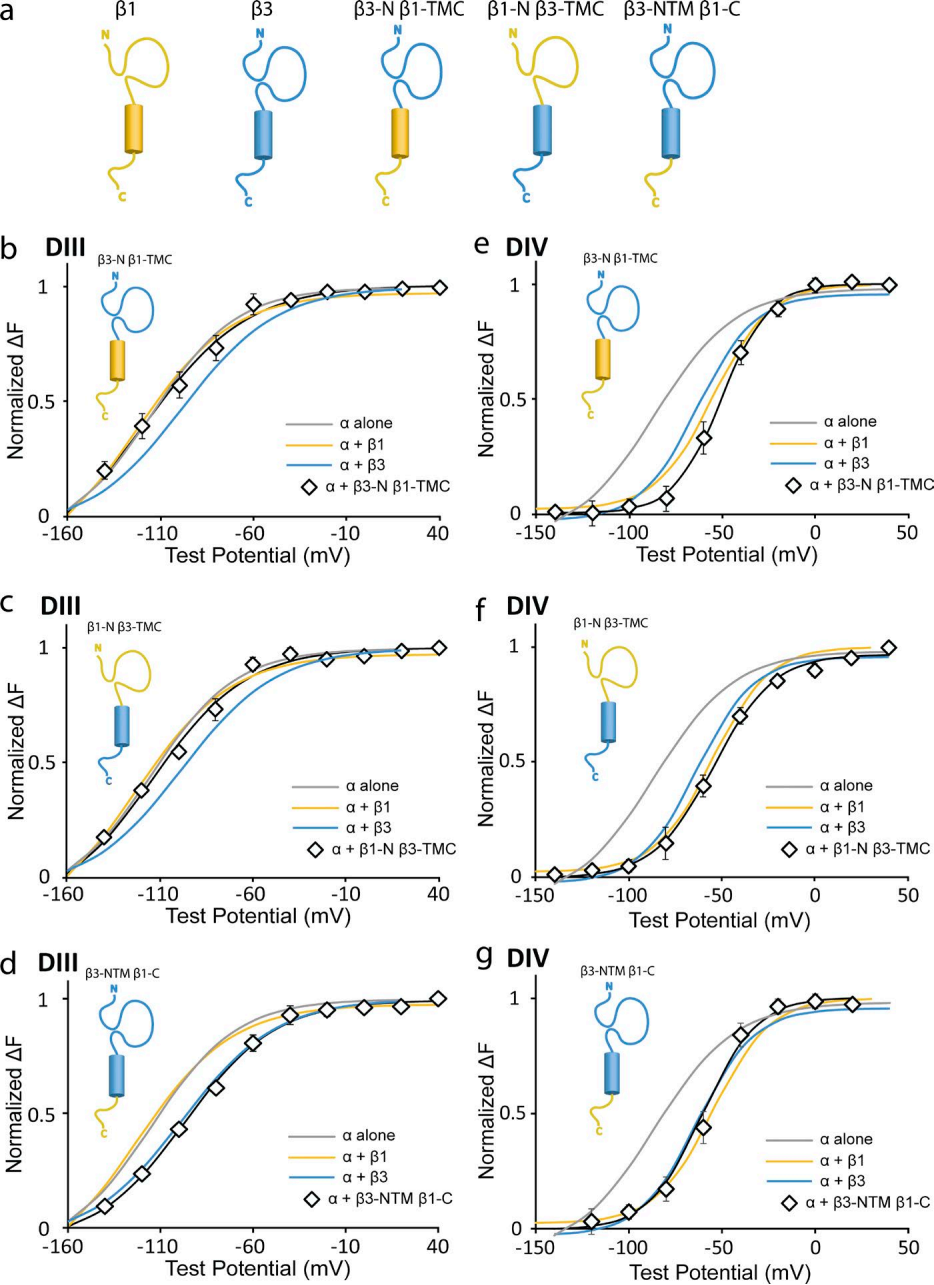
**β1/β3 chimeras reveal that both the extracellular and transmembrane domains of β3 are essential for its modulation of DIII VSD.** Ionic currents and fluorescence signals are acquired as shown in Fig. 1. The mean ± SEM is reported for groups of three to five cells. F-V curves for WT β1 and β3 are shown as Boltzmann fit curves because they have been previously shown in Figs. 1 and 2. (a) Three β1/β3 chimeras were created—β3-N β1-TMC, β1-N β3-TMC, and β3-NTM β1-C—based on predicted extracellular, transmembrane, and intracellular sequences from Uniprot. mRNA encoding β1/β3 chimeras and DIII or DIV VCF constructs were coinjected at a molar ratio of 3:1. (b–d) DIII F-V curve with β1 (yellow line), β3 (blue line), β1/β3 chimera (black diamonds), or without β (gray line). (b) DIII F-V with β3-N β1-TMC chimera resembles DIII F-V of channel with β1 or α alone. (c) DIII F-V with β1-N β3-TMC chimera resembles DIII F-V of channel with β1 or α alone. (d) DIII F-V with β3-NTM β1-C chimera overlaps with DIII F-V of channel with β3. Boltzmann fit parameters are listed in Table 3. (e–g) DIV F-V curve with β1 (yellow line), β3 (blue line), β1/β3 chimera (black diamonds), or without β (gray line). (e) β3-N β1-TMC chimera induces a bigger shift in DIV F-V compared with β1 or β3. (f and g) β1-N β3-TMC and β3-NTM β1-C chimeras resemble DIV F-V of channels with β1 or β3. Boltzmann fit parameters are listed in Table 3.

We observed that β1 and β3 have distinct interactions with channel VSDs, especially the DIII-VSD. In response, we sought to further understand which part of the β subunit is essential for these interactions. We created three chimera β1 and β3 subunits (Fig. 6 a): one with the β3 extracellular domain and β1 transmembrane and intracellular domain (β3-N β1-TMC), one with the β1 extracellular domain and β3 transmembrane and intracellular domain (β1-N β3-TMC), and one with both the extracellular and transmembrane domains of β3 and the β1 intracellular domain (β3-NTM β1-C). Comparison of the DIII F-V curves for all three chimeras illustrates that both the extracellular and transmembrane domains of β3 are necessary for its modulation of the DIII-VSD activation because only the chimera that contains both the extracellular and transmembrane domains of β3 (β3-NTM β1-C) caused a depolarizing shift in the DIII F-V curve (Fig. 6 d and Table 3) compared with α alone (ΔV_1/2_ = 22.9 ± 5.0 mV, P = 0.003), resembling the shift induced by the WT β3 subunit (ΔV_1/2_ = 20.7 ± 3.9 mV, P = 0.01). The other two β1/β3 chimeras caused no shift in the DIII F-V curve compared with α alone, resembling the β1 subunit effects (Fig. 6, b and c; and Table 3).

**Table 3.** Parameters of Boltzmann fit to DIII and DIV F-V curves for VCF constructs expressed with β1/β3 chimeras

**Parameter**	**DIII + β3-N β1-TMC**	**DIII + β1-N β3-TMC**	**DIII + β3-NTM β1-C**
**DIII F-V**			
V_1/2_	−120.8 ± 10.6	−112.6 ± 3.1	−95.0 ± 3.3
k [n]	27.3 ± 3.5 [5]	24.6 ± 1.9 [4]	22.9 ± 0.7 [3]
	**DIV+ β3-N β1-TMC**	**DIV+ β1-N β3-TMC**	**DIV+ β3-NTM β1-C**
**DIV F-V**			
V_1/2_	−50.5 ± 3.0	−55.2 ± 2.9	58.5 ± 1.8
k [n]	12.5 ± 0.7 [4]	16.4 ± 1.4 [3]	12.5 ± 2 [3]

The DIV-VSD response to these β1/β3 chimeras is more difficult to interpret because both WT β1 and β3 cause comparable depolarizing shifts in the DIV F-V curve (Figs. 1 and 2). Notably, the β3-N β1-TMC chimera causes a larger depolarizing shift of the DIV-VSD (ΔV_1/2_ = 32.7 ± 1.2 mV, P = 0.001) than β1 or β3 (Fig. 6 e). Both β1-N β3-TMC and β3-NTM β1-C cause DIV F-V depolarization that is similar to that of WT β1 or β3 (Fig. 6, f and g; and Table 3). This result suggests that the interaction of the β3 extracellular domain with the channel can cause additional modulation of the DIV-VSD and induce an interaction mechanism that is distinct from the transmembrane and C terminus of the β1 subunit. Previously, we observed that the WT β1 subunit causes the DIV-VSD to deactivate more quickly (Fig. 1). Comparing the DIV-VSD deactivation kinetics of these chimeras, we note that both the β3-N β1-TMC and β3-NTM β1-C chimeras that contain the β1 C terminus resemble the fast DIV deactivation induced by WT β1 (β3-N β1-TMC: t_100–10%_ = 3.9 ± 0.6 ms, P = 0.0002; β3-NTM β1-C: t_100–10%_ = 3.7 ± 0.3 ms, P = 0.0004, compared with α alone; Fig. S5 and Table 4), suggesting that the β1 C terminus is important for speeding DIV-VSD deactivation upon membrane repolarization.

**Table 4.** DIV VSD deactivation rate, measured by the time when 90% of fluorescence recovered to resting level (t_100–10%_) measured at 0 m**V**

**Parameter**	**DIV alone**	**DIV + β1**	**DIV + β3**	**DIV + β3-Nβ1-TMC**	**DIV + β1-N β3-TMC**	**DIV + β3-NTM β1-C**
t_100–10%_	13.2 ± 0.4	4.5 ± 0.7	11.5 ± 1.4	3.9 ± 0.6	7.2 ± 0.1	3.7 ± 0.3

By monitoring DIII and DIV VSD conformations in the presence of β1/β3 chimeras, we found that the extracellular and transmembrane domains of the β3 subunit are essential for depolarizing DIII-VSD activation, which controls channel current activation kinetics. The C terminus of β1 is essential for speeding DIV-VSD deactivation kinetics upon membrane repolarization, which will affect channel recovery from inactivation.

### β1 and β3 effects on the DIII and DIV VSDs are not independent

**Figure 7.**
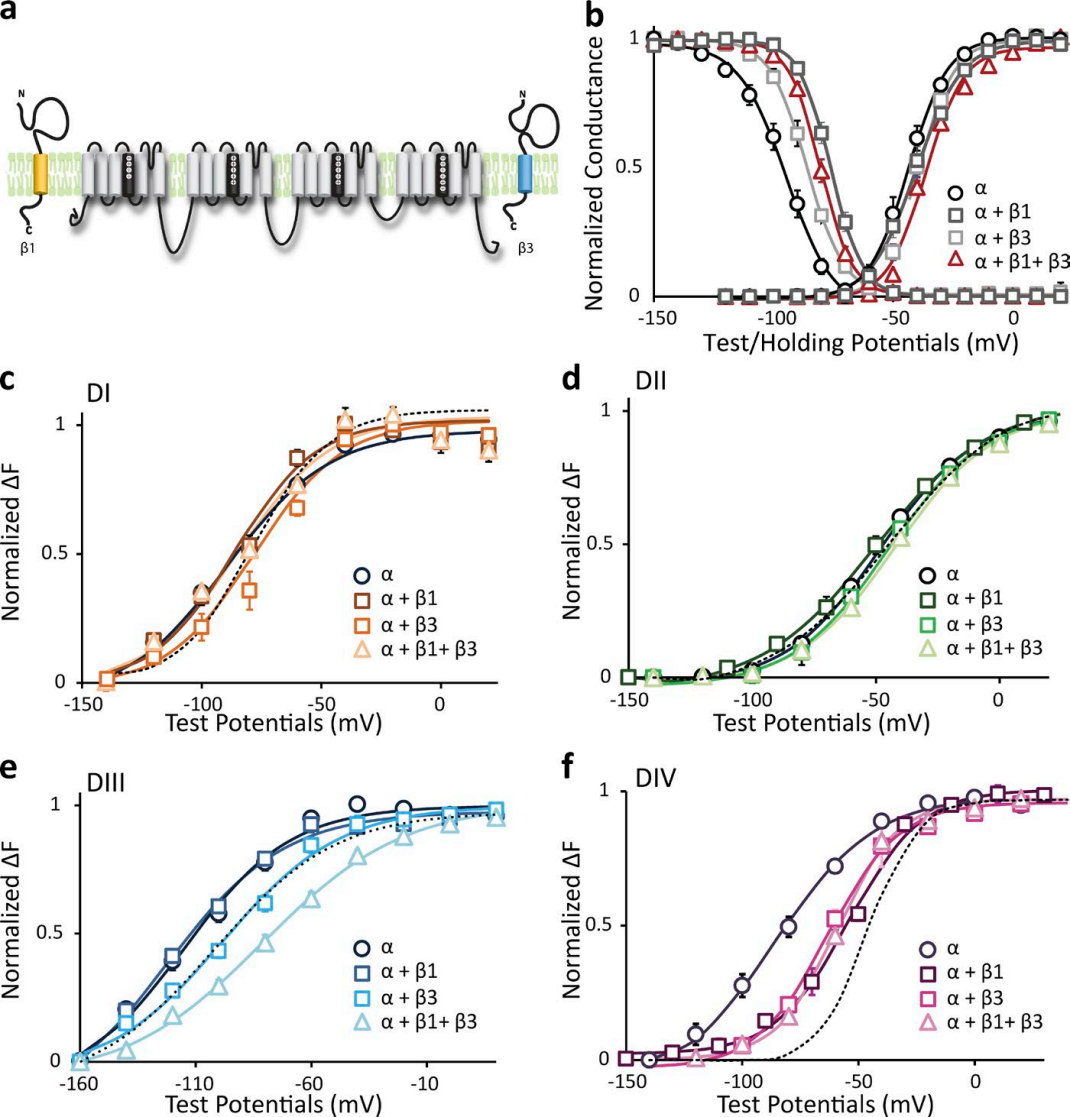
**Simultaneous coexpression of the β1 and β3 subunits reveals cooperativity between these two subunits.** (a) Both β1 and β3 subunits are coexpressed with Na_V_1.5 by coinjecting mRNAs encoding Na_V_1.5, β1, and β3 at a molar ratio of 1:3:3. Ionic currents and fluorescence signals are acquired as shown in Fig. 1. Mean ± SEM is reported for groups of three to six cells. (b) Voltage dependence of activation (G-V) and steady-state inactivation (SSI) for LFS-DIII Na_V_1.5 coexpressed with β1 (dark gray squares), β3 (light gray squares), β1+ β3 (triangles), or without β (circles). For coexpression of both β1 and β3, the molar ratio of β1:β3 is 1:1. (c–f) DI–DIV F-V curve with β1 (dark squares), β3 (light squares), β1+ β3 (triangles), or without β (circles) coexpressed. The dotted line represents the sum of the shifts induced by β1 and β3 with respect to no β. (c) β1+ β3 coexpression resembles the DI F-V of α alone. (d) β1+ β3 coexpression induces a very small shift in DII F-V compared with α alone. (e) β1+ β3 coexpression produces stronger depolarization of DIII F-V than the linear addition of the β1 and β3 effects. (f) β1+ β3 coexpression resembles the DIV F-V depolarization effect induced by β1 or β3 alone.

Both β1 and β3 have been shown to express in the ventricular myocardium (Fahmi et al., 2001; Olesen et al., 2011) to modify Na_V_1.5 gating in concert. To assess the molecular consequences when both β1 and β3 subunits are expressed, we coinjected mRNA of the α, β1, and β3 subunits at a molar ratio of 1:3:3 (Fig. 7 a). As with coexpression of β1 or β3, simultaneously coexpressing the channel with both causes a depolarizing shift in the SSI curve (Fig. 7 b) compared with the α subunit alone. The magnitude of this shift does not significantly differ from the shifts induced by β1 or β3 (Fig. 7 b), raising two possible mechanisms. One explanation is that only one of the β1 and β3 subunits can coassemble with the α subunit at a time, so that the shift is a mean of the shifts induced by either β1 or β3. The other possibility is that β1 and β3 binding to the α subunit is not exclusive, and there might be some cooperation and interaction between the β1 and β3 subunits that are bound to the same α subunit. The latter mechanism appears to be more likely, as we also observed a depolarizing shift in the G-V curve caused by coexpression with both the β1 and β3 subunits (ΔV_1/2_ = 7.2 ± 2.3 mV, P = 0.02; Fig. 7 b), which was not seen in α with only β1 or β3.

To gain insight into the potential interaction mechanisms between β1 and β3, we assessed the coexpression of both β1 and β3 on the activation of each VSD. Coexpression of β1 and β3 has minimal effects on DI and DII VSD activation, as no significant shift was observed in the DI and DII F-V curve (Fig. 7, c and d), consistent with the previous observation that β1 and β3 do not perturb DI or DII VSDs. Compared with α alone, coexpression with both β1 and β3 caused a depolarizing shift in the DIII-VSD (Fig. 7 e). The magnitude of this shift was greater than the linear addition of the shifts induced by β1 only and β3 only (dotted line), suggesting that when both β1 and β3 were present, they produced additional depolarization of DIII-VSD activation, corresponding to the depolarizing shift in channel activation. Thus, we infer that there must be cooperation between β1 and β3 that causes a greater effect on the DIII VSD when both are interacting with the channel. 

In contrast, expressing β1 and β3 together caused a DIV F-V depolarizing shift that most closely resembles the shift produced by β1 or β3 only (Fig. 7 f) and that also corresponds to the SSI shift (Fig. 7 b). If β1 and β3 act independently, the DIV F-V shift should resemble the linear addition of β1- and β3-induced shifts (Fig. 7 f, dotted line). Thus, this result suggests that β1 and β3’s modulation of the DIV-VSD is not additive.

Together, these results show that β1 and β3 binding to the Na_V_1.5 channel is not exclusive, and when both β subunits are present, their cooperativity further depolarizes DIII-VSD activation, which leads to additional modifications of channel activation. The physiological impact of this result is limited because this experiment was conducted in the condition of overexpressing β subunits, which may cause additional binding that may not happen in native cells. More information regarding the stoichiometry of α, β1, and β3 in native cells is required to infer the physiological consequences of β1 and β3 subunit cooperativity.

## Discussion

The noncovalently bound Na_V_ channel β1 and β3 subunits were first identified in 1985 (Messner and Catterall, 1985) and 2000 (Morgan et al., 2000). Despite recent findings showing that these subunits play a critical role in regulating neuronal and cardiac electrophysiology (Calhoun and Isom, 2014), the precise mechanisms that they use to modulate channel gating have not been described. In this study, we used VCF to test the hypothesis that the β1 and β3 subunits regulate Na_V_ channel kinetics via the VSDs. We discovered that WT β1 subunit coexpression shifts DIV-VSD activation to depolarized potentials, consistent with the shift in SSI. β1 subunits also relieve the immobilization of the DIII and DIV VSDs by fast inactivation, which potentially contributes to the increased rate of channel recovery from inactivation induced by β1.

The WT β3 subunit also regulates channel inactivation with a corresponding shift in DIV-VSD activation. However, in contrast to β1, there is a prominent WT β3 interaction with the DIII-VSD. We believe that β3 modulation of the DIII-VSD is primary because we showed that the transmembrane domain of β3 is very close to the DIII S4 by tryptophan-induced fluorophore quenching. Moreover, β1/β3 chimeras mainly affect the DIII-VSD without changing DIV-VSD activation. Recently, we showed that DIII-VSD deactivation strongly correlates with channel recovery from inactivation, a phenomenon that is determined by the DIII S4–S5 linker’s interaction with the inactivation gate after depolarizing pulses of ∼100 ms (Hsu et al., 2017). Here, we observed that β3 does not affect channel recovery from inactivation even though it speeds DIII-VSD deactivation, suggesting that β3 possibly disrupts the interaction between the DIII S4–S5 linker and the inactivation gate, abolishing DIII-VSD regulation of recovery from inactivation.

Despite the β1 and β3 subunits being homologous, we demonstrate that they have distinct interactions with the Na_V_ channel VSDs, resulting in different current kinetics and rates of inactivation recovery. Consistently, our results from the tryptophan-induced quenching experiment showed that β1 and β3 assemble with the channel at different locations. As β1 and β3 have very different spatial and temporal expression patterns in the heart (Domínguez et al., 2005; Okata et al., 2016), the molecular interactions that we have observed will significantly affect their regulation of tissue excitability. For regions that have higher β1 expression—for example, Purkinje fibers in the heart (Domínguez et al., 2005)—we would expect cells to be more excitable because β1 causes Na_V_ channels to recover more quickly and increase channel availability, consistent with the Purkinje fiber role of fast conduction of cardiac excitation. During heart development, β1 expression increases, whereas β3 expression decreases (Domínguez et al., 2005; Okata et al., 2016). This dynamic temporal expression pattern suggests that β1 contributes more to mature Na_V_ channel properties, such as increased excitability. Thus, we expect that molecular-level differences in β1 and β3 regulation of the DIII and DIV VSDs will affect organ-level behavior.

### β1 and β3 localization within the Na_V_1.5 channel complex

The β1 subunit has been found to express and coassemble with both neuronal and cardiac Na_V_ channels (Isom et al., 1992). There is a general consensus that when β1 coassembles with Na_V_1.5, it regulates SSI (Calhoun and Isom, 2014). However, the direction and magnitude of the inactivation shift vary depending on the expression system used and the protocols applied (Calhoun and Isom, 2014). This variability may be linked to the interaction between β1 and other members of the macromolecular Na_V_ channel complex in native cells, such as ankyrin G (Malhotra et al., 2002). Thus, the nature of α–β1 interaction may vary by expression system, precluding the identification of a universal phenotype.

**Figure 8.**
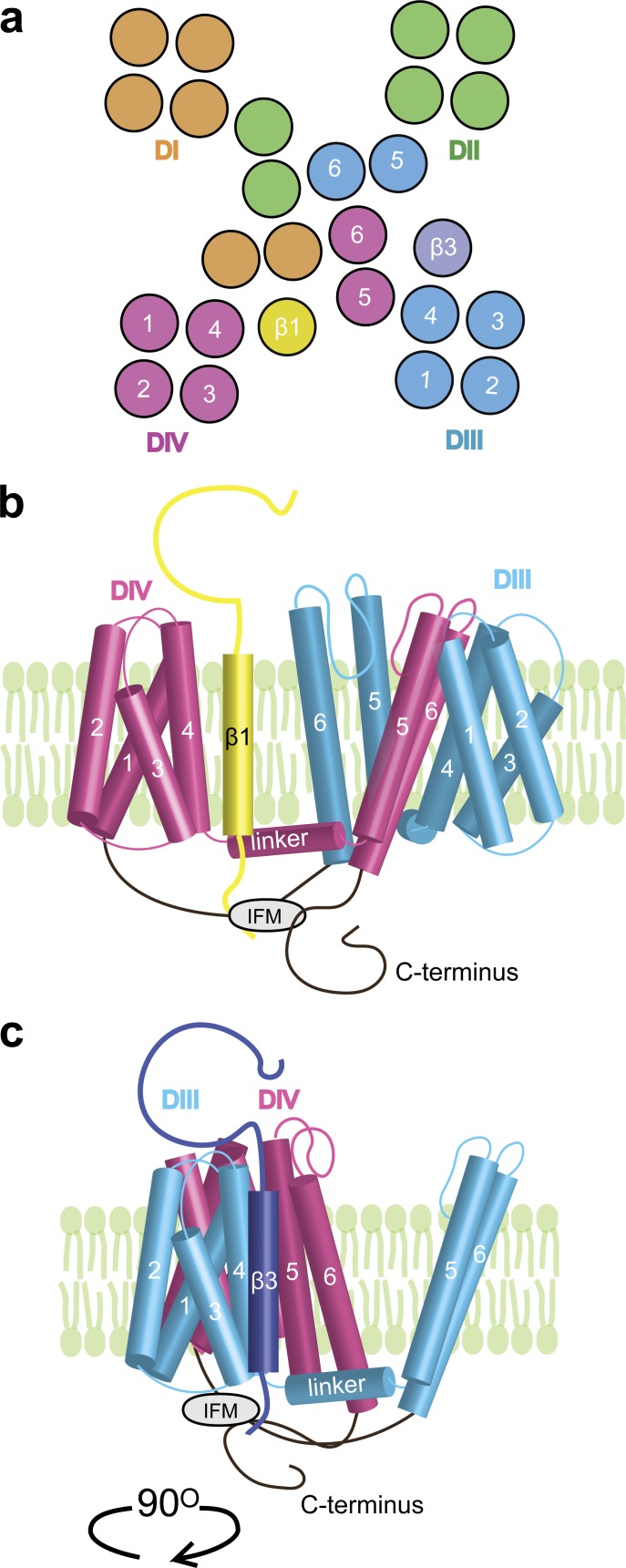
**Proposed model for β1 and β3 assembly with Na_V_1.5 channel.** (a) Extracellular view of the Na_V_1.5 channel based on the Na_V_ab structure (Payandeh et al., 2011). Each domain is color-coded as shown in Figs. 1, 2, 3, 4, 5, and 6. The β1 and β3 locations suggested by our results are shown. (b) Side view of Na_V_1.5 coassembled with β1 subunit. Only DIII (blue) and DIV (pink) are shown for clarity. Our model suggests that β1 is located in the cleft between the DIII VSD and DIV VSD, allowing it to interact with the DIV VSD, the C terminus, and potentially the S4–S5 linker of DIV to modify DIV VSD movements. (c) Side view of Na_V_1.5 (rotated 90°) coassembled with β3 subunit. Our model suggests that β3 is located in the cleft between the DIII VSD and DII VSD, next to the S4 segment of DIII, allowing it to strongly modify DIII VSD activation and affecting DIII VSD–pore coupling by interacting with the hinge connecting the S4 and S4–S5 linker of DIII.

Several α–β1 interaction sites have been proposed. On the α subunit, a C-terminal mutation was able to eliminate β1 regulation of Na_V_1.1 current kinetics (Spampanato et al., 2004), and Na_V_1.4/Na_V_1.5 chimeras show that the S5–S6 linker of DIV plays a role in α–β1 interaction (Makita et al., 1996). Both results suggest that binding occurs near the DIV domain. Aside from the consequences of direct binding, the β1 subunit has also been shown to introduce the surface charges that electrostatically affect channel gating (Ferrera and Moran, 2006). We observed that WT β1 mainly affects the voltage dependence of DIV-VSD activation and its deactivation kinetics through possible direct interaction with the DIV-VSD, which suggests β1 proximity to the DIV-VSD. Together, these results support the hypothesis that β1 modulates inactivation by altering DIV-VSD activation. We infer that β1 most likely resides in the cleft between the DIII and DIV VSDs (Fig. 8, a and b).

The β3 subunit is homologous to β1 and also interacts with Na_V_ channels noncovalently. Less is known about the α–β3 interaction. Because the β1 and β3 subunits are homologous (50%), it has been generally supposed that β3 interacts with the channel via the same mechanism as β1 (Namadurai et al., 2015). In contrast, we found that the WT β3 subunit caused a large depolarizing shift in DIII-VSD activation in addition to the DIV shift. The depolarization of DIII-VSD activation slowed down ionic current activation and inactivation kinetics, allowing the DIII-VSD to play a more prominent role in regulating this gating over a physiological range of potentials. Further, we demonstrated β3 proximity to the DIII S4 segment, as we observed that a tryptophan mutation on top of the β3 subunit strongly quenches the fluorophore attached to the DIII S3–S4 linker. It is most likely that β3 is adjacent to the DIII S4 segment in the cleft between the DII and DIII VSDs, which allows β3 to directly interact with the DIII-VSD. Altered DIII-VSD activation can then allosterically affect DIV-VSD activation. Yet, we observed that β3 still depolarized the DIV-VSD when the DIV VSD–pore coupling via the S4–S5 linker was abolished by the N1759A mutation, suggesting that this coupling to the DIV VSD takes place via alternative mechanisms, such as the DIII–DIV linker.

The importance of β3 in maintaining normal cardiac function has been highlighted by *scn3b* knockout mice. These mice exhibit slowed sinoatrial and atrioventricular conduction, burst pacing–induced atrial tachycardia, fibrillation, and ventricular tachycardia (Hakim et al., 2008, 2010). Consistent with our results (Fig. 2), knocking out β3 shifts Na_V_ SSI to negative potentials, reducing peak Na^+^ current in the ventricle, which causes slowed conduction and decreased action potential duration in the endocardium and epicardium. Given the consistency of the knockout mouse phenotype with our results, we infer that β3 regulation of the Na_V_1.5 DIII and DIV VSDs significantly determines action potential morphology and conduction.

When we expressed β1 and β3 together, we observed enhancement of the depolarizing DIII-VSD shift and an exclusive effect of β1 on the DIV-VSD (Fig. 6). These results imply that the subunits do not interact with the channel independently. Previous work has shown that heterophilic interaction between the β1 and β3 subunits can occur via their respective Ig domains (Yereddi et al., 2013), which could possibly alter their interaction with each VSD. β3 binding to β1 may affect its interaction with the DIII-VSD. Our proposed localization of β1 and β3 would bring the extracellular domains of both subunits close to the DIII S4 (Fig. 8). In sum, our results show that both the β1 and β3 subunits are likely able to coassemble with a single Na_V_1.5 channel complex and that this coassembly significantly affects channel function.

### Each domain of β1 and β3 has distinct interactions with the Na_V_1.5 channel

By measuring VSD conformation in the presence of β1/β3 chimeras, we showed that both the extracellular and transmembrane domains of β3 are necessary for β3 depolarization of the DIII-VSD. When channels were coexpressed with β1/β3 chimeras containing the β3 extracellular domain and the β1 transmembrane and intracellular domain, DIII-VSD activation was not depolarized, suggesting that the transmembrane domain of β3 is critical for localizing the β3 subunit to this location in Na_V_1.5, allowing the extracellular Ig domain of β3 to interact with the DIII-VSD. We also showed that the C terminus of the β1 subunit is necessary for relieving DIV-VSD immobilization and that the β3 C terminus is important for relieving DIII-VSD immobilization from fast inactivation (Fig. S5, c and e). Previously, we showed that the interaction between the fast inactivation gate (IFM) and the N1659 residue on the DIV S4–S5 linker plays an important role in immobilizing the DIII and DIV VSD. It is plausible that the β1 C terminus can interact with the intracellular DIV S5 segment, altering this interaction. These results show that β1 and β3 could interact with the Na_V_ channel through multiple interaction sites and that both the extracellular domain and the C terminus play important roles in determining their gating properties.

### Two-step movements of DIII VSD revealed by high expression of β3

As with K_V_ channels, two-step transitions of Na_V_ channel VSDs have been proposed based on previous observations that pulses of increasing duration alter the DIII-VSD deactivation rate (Varga et al., 2015; Hsu et al., 2017). Our results support this suggestion by showing that high levels of β3 subunit expression cause two prominent components of DIII VSD activation. This phenomenon is similar to KCNQ1 channels that show two-step activation in the presence of KCNE1, which stabilizes the activated VSD closed-pore state and slows ionic current activation (Barro-Soria et al., 2014). Similarly, the activation of Na^+^ current is slower when β3 separates the DIII-VSD transition into two components. Additionally, because Na_V_ channels have very fast and prominent inactivation, the separation of DIII-VSD movement will increase the amplitude of the Na^+^ current as the channels are excited repetitively at a relatively high frequency (>3 Hz).

Our study shows that β1 and β3’s differential interactions with the DIII and DIV VSDs determine their regulation of Na^+^ current and cell excitability. These distinct regulatory mechanisms are essential for understanding how β subunits regulate excitable cells and how mutant β subunits cause disease.

## Supplementary Material

Supplemental Materials (PDF)
