# Supplementary material for: Mechanisms of noncovalent β subunit regulation of NaV channel gating
Source: J Gen Physiol. 2017 Aug 7;149(8):813–31. doi: 10.1085/jgp.201711802 (PMC5560778; doi:10.1085/jgp.201711802)
Supplement: Supplemental Materials (PDF) [file JGP_201711802_sm.pdf]

## SUPPLEMENTAL MATERIAL

Zhu et al., https://doi.org/10.1085/jgp.201711802

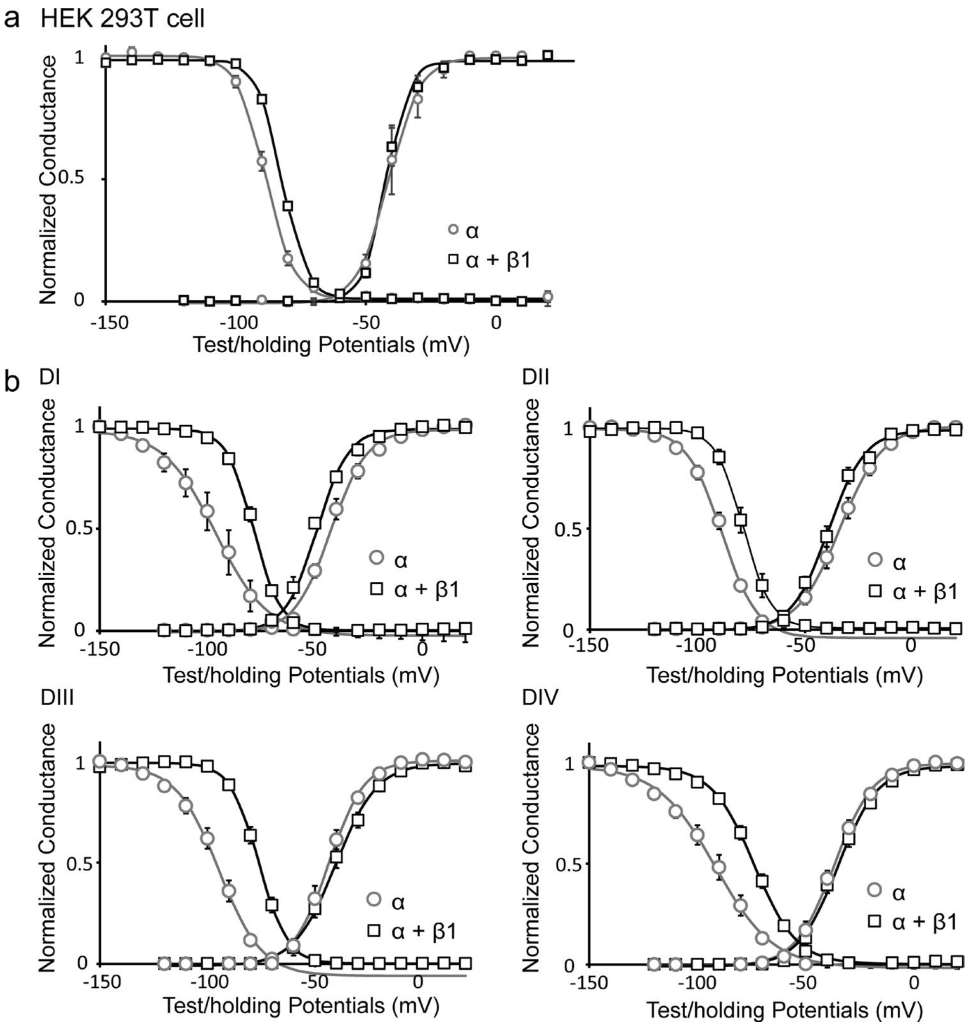


Figure S1. **Steady-state activation (G-V) and steady-state inactivation (SSI) for $Na_V1.5$ with or without β1 expressed in HEK293T cells and for four VCF constructs with or without β1.** The mean ± SEM is reported for groups of 3–19 cells. (a) HEK293T cells were transfected with cDNAs encoding $Na_V1.5$ (α) and β1 at a 1:1 ratio. Currents were recorded using a whole-cell patch clamp. Voltage dependence of activation (G-V) and SSI for WT $Na_V1.5$ with β1 (α + β1, squares) or without β1 (α, circles). The same protocols were used as shown in Fig. 1. (b) Voltage dependence of activation (G-V) and SSI for four VCF constructs (DI-V215C, DII-S805C, DIII-M1296C, and DIV-S1618C) with β1 (α + β1, squares) or without β1 (α, circles). Data are fit with a Boltzmann equation (solid lines; see Materials and methods), and parameters are reported in Table 1.



The Journal of General Physiology

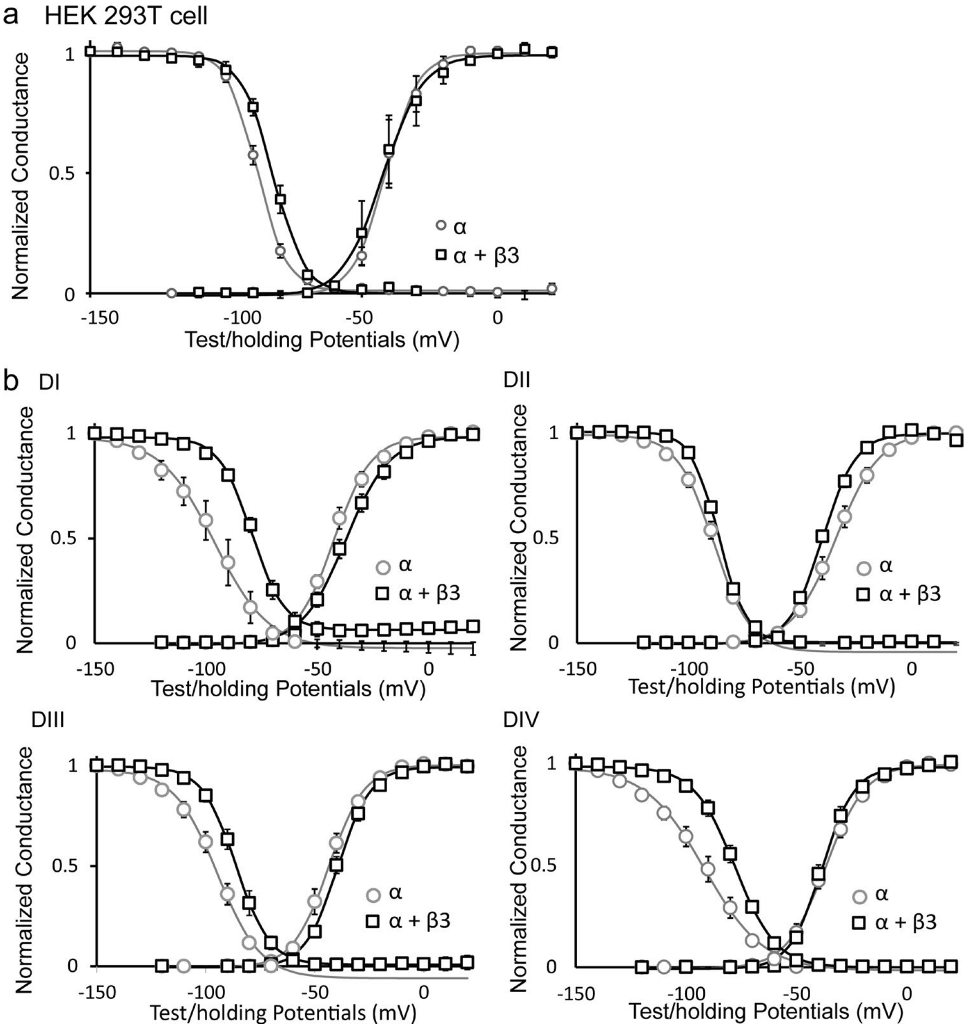


Figure S2. **Steady-state activation (G-V) and steady-state inactivation (SSI) for $Na_V1.5$ with or without β3 expressed in HEK293T cells and for four VCF constructs with or without β3.** The mean ± SEM is reported for groups of 3–19 cells. (a) HEK293T cells were transfected with cDNAs encoding $Na_V1.5$ (α) and β3 at a 1:1 ratio. Currents were recorded using a whole-cell patch clamp. Voltage dependence of activation (G-V) and SSI for WT $Na_V1.5$ with β3 (α + β3, squares) or without β3 (α, circles). The same protocols were used as shown in Fig. 1. (b) Voltage dependence of activation (G-V) and SSI for four VCF constructs (DI-V215C, DII-S805C, DIII-M1296C, and DIV-S1618C) with β3 (α + β3, squares) or without β3 (α, circles). Data are fit with a Boltzmann equation (solid lines), and parameters are reported in Table 1.

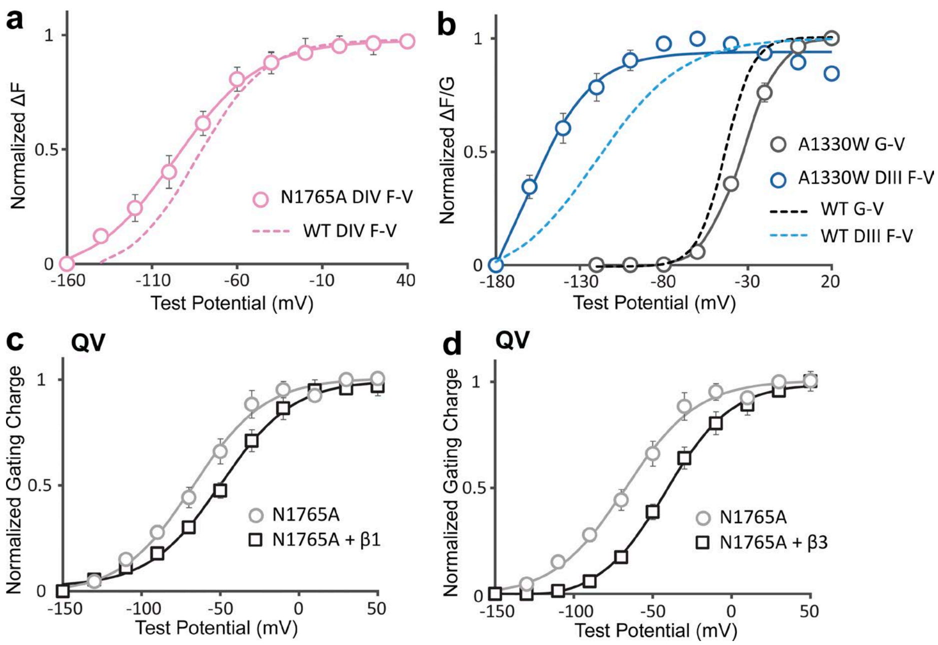


Figure S3. **Voltage dependence of fluorescence and gating charges for decoupling mutations A1330W and N1765A.** (a) DIV F-V curves of the N1765A channel compared with those of the WT channel (dashed line). DIV activation is not significantly altered by N1765A mutation. (b) DIII F-V curve and channel G-V curve for the A1330W channel compared with the WT channel (dashed lines). A1330W greatly hyperpolarizes DIII VSD activation without significantly affecting channel activation (G-V) compared with WT. (c) Q-V curves for the N1765A channel with β1 (black squares) or without β1 (gray circles). β1 still induces a depolarizing shift in the Q-V curve of the N1765A channel. (d) Q-V curves for the N1765A channel with β3 (black squares) or without β3 (gray circles). β3 also induces a depolarizing shift in the Q-V curve of the N1765A channel.

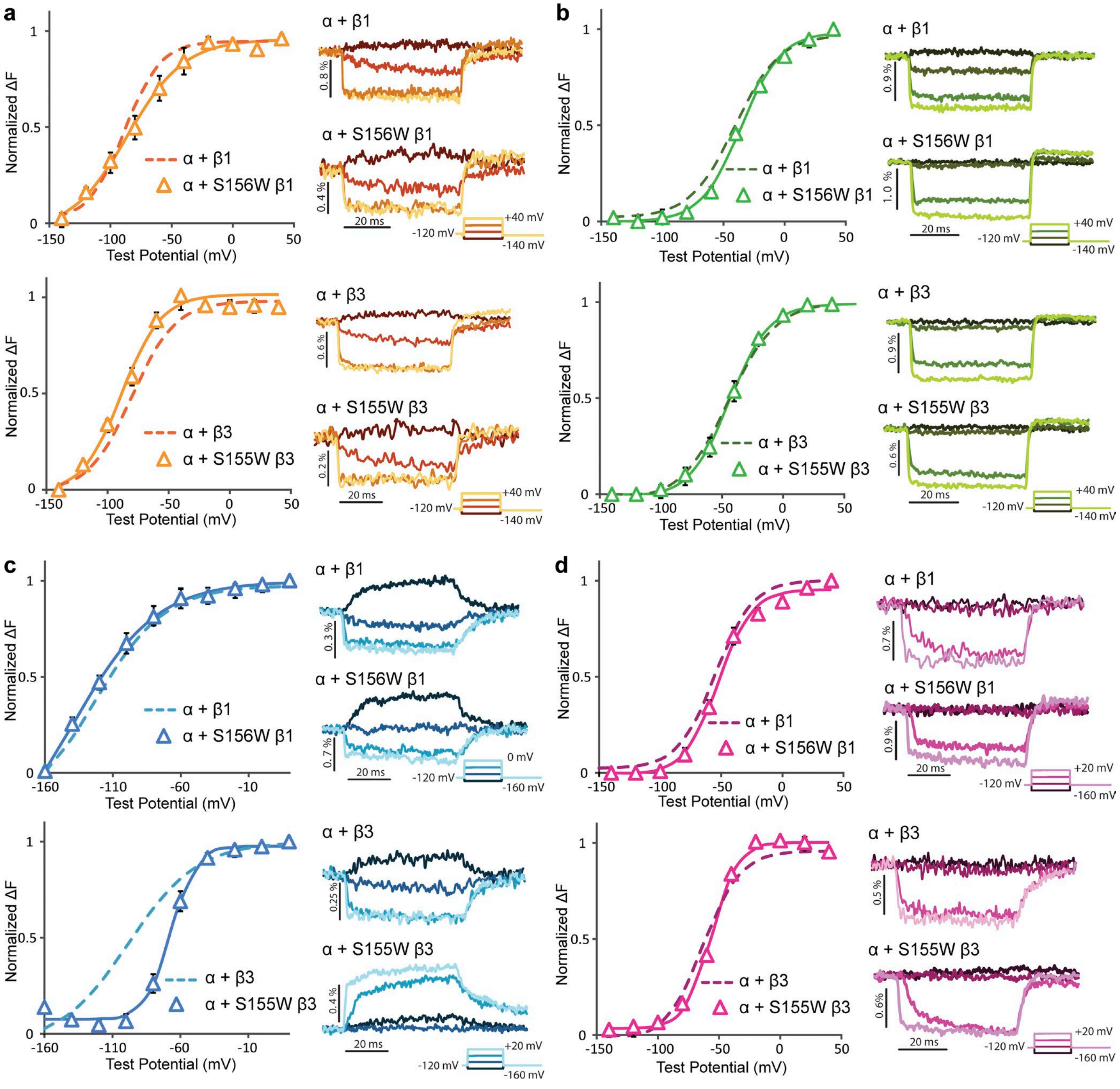


Figure S4. **F-V curves and representative fluorescence traces of DI–DIV LFS coexpressed with S156W β1 or S155W β3.** DI (a), DII (b), DIII (c), and DIV (d) F-V and fluorescence traces with S156W β1 or S155W β3 compared with WT β1 or β3. The only curve that is significantly altered is that of the DIII VSD in the presence of S155W β3.

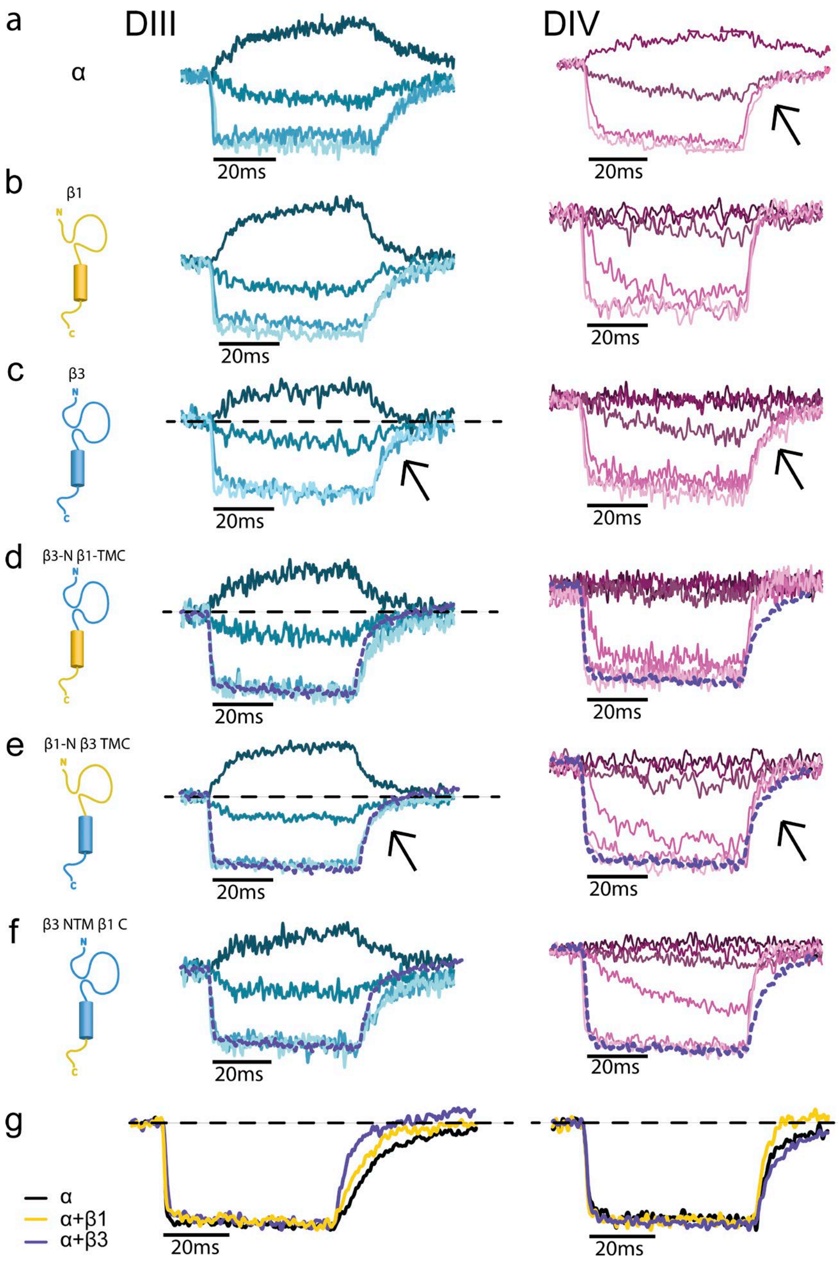


Figure S5. **Comparison of DIII (blue) and DIV (pink) fluorescence kinetics of channels without β, with β1 or β3, and three β1/β3 chimeras.** DIV deactivation rates are reported in Table 4. (a–c) Representative DIII (blue) and DIV (pink) fluorescence traces for α alone, α + β1, and α + β3. (a) When channels are expressed without β subunits, both DIII and DIV VSD deactivation is slow. (b) β1 speeds up both DIII and DIV VSD deactivation, particularly DIV. (c) β3 speeds up DIII VSD deactivation without affecting DIV kinetics. (d–f) Representative DIII (blue) and DIV (pink) fluorescence traces for β1/β3 chimeras. Mean fluorescence trace of DIII with β3 (dotted purple line) is overlaid to compare the deactivation kinetics of (d) β3-N β1-TMC, (e) β1-N β3-TMC, and (f) β3 NTM β1-C. (d) β3-N β1-TMC causes very similar DIII and DIV fluorescence kinetics as WT β1. (e) β1-N β3-TMC has similar DIII and DIV fluorescence kinetics as WT β3. (f) β3-NTM β1-C has similar DIII and DIV deactivation kinetics as WT β1. Overall, the chimeras that contain the β1 C terminus have fast DIV VSD deactivation, similar to WT β1, whereas chimeras containing the β3 C terminus have DIII and DIV VSD kinetics similar to that of WT β3, suggesting the importance of the C terminus of β subunits in regulating DIII and DIV VSD deactivation kinetics. (g, left) DIII and (g, right) DIV fluorescence traces during 0-mV pulse for α alone (black), α + β1 (yellow), and α + β3 (purple) were overlaid to show differences in deactivation kinetics. Each fluorescence trace is shown as a mean of two normalized fluorescence traces.
